# Evaluating the Needs and Characteristics of Individuals of Low Socioeconomic Status Using Digital Health Technology to Address Health-Related Social Needs: Mixed Methods Study With Patients and Care Providers

**DOI:** 10.2196/69545

**Published:** 2025-09-12

**Authors:** Kayla Li Haydon, Cynthia LeRouge, Polina Durneva, Maria Paula Diaz Campo, David Richard Brown

**Affiliations:** 1 Herbert Wertheim College of Medicine Florida International University Miami, FL United States; 2 School of Public Health and School of Medicine University of Washington Seattle, WA United States; 3 Department of Information Systems and Business Analytics Loyola Marymount University Los Angeles, CA United States; 4 Jarvis College of Computing and Digital Media DePaul University Chicago, IL United States

**Keywords:** social determinants of health, health-related social needs, consumer health informatics, mobile health, digital inclusion

## Abstract

**Background:**

Social determinants of health (SDOH) are the conditions in which people are born, grow, live, work, and age, encompassing social and economic factors that shape health outcomes. There is an increasing call to leverage digital health technology (DHT) to address SDOH and health-related social needs and establish connections to resources and services.

**Objective:**

This study aimed to (1) identify the DHT-related characteristics of DHT users with low socioeconomic status (SES), (2) determine the needs and preferences of DHT users with low SES, and (3) explore how current SDOH-DHT addresses these needs and preferences in addressing their health-related social needs.

**Methods:**

We used a multiphase, mixed method, user-centered design approach. In phase 1, we developed a user profile based on a literature review, aggregate data, interviews with 26 low-SES individuals, and focus groups with 28 professionals. In phase 2, we conducted a landscape analysis of 17 existing SDOH-DHTs.

**Results:**

DHT users of low SES had diverse social and technology characteristics. Five key themes emerged regarding user needs and preferences: (1) user-centered design, including multilingual support, visual guidance, and customization; (2) efficient, solution-based assessment of social risks, assets, and needs; (3) e-caring support features; (4) user education and feedback mechanisms; and (5) trust, privacy, and security. The landscape analysis revealed that current SDOH-DHT features do not adequately meet these needs.

**Conclusions:**

Discrepancies between target user needs and current DHT features represent missed opportunities in developing user-centered tools for individuals of low SES. Findings underscore the importance of inclusive, empowering, and responsive design in SDOH-DHT to bridge health disparities and advance public health.

## Introduction

### Background

Social determinants of health (SDOH) are “the conditions in which people are born, grow, live, work, and age,” encompassing social and economic factors that shape health outcomes [1]. These determinants can function as assets or risk factors, with individual-level adverse social conditions being defined as social risk factors [2]. “Health-related social needs” (HRSN) differ from social risk factors by reflecting both the presence of risks and individuals’ preferences for addressing them [2]. These immediate needs, such as food insecurity and social isolation, stem from upstream SDOH, including economic stability, education, health care access, and the physical environment [3]. The resulting social gradient creates a corresponding health gradient with particular impact on lower socioeconomic status (SES) populations [4-6].

Studies have highlighted the impact of SES on both health outcomes and technology use, underscoring the importance of considering SES in health technology research [4,7,8]. A broad definition of “low SES” includes a lack of resources (eg, education and income) or measures of poverty (eg, food insecurity and income-to-needs ratio) [9]. Individuals of low SES often lack the knowledge or resources to respond to social needs and maintain health [6]. Bourdieu’s theory of capital—economic, social, and cultural—provides a useful framework for understanding how these determinants operate [10]. Economic capital refers to financial resources, social capital to networks and relationships, and cultural capital to knowledge and dispositions that confer social value. Each form of capital can impact health outcomes and access to health care [11]. Unmet social needs are closely associated with disparities in health outcomes, quality of life, and life expectancy, disproportionately affecting marginalized communities [12]. While SES, typically measured by income, education, and occupation, is a key social determinant of health, SDOH encompasses a broader range of social and environmental conditions beyond individual-level SES [9]. SES and related SDOH significantly impact health outcomes, with studies estimating that they contribute to around half of health outcome variation, while health behaviors account for about a third, and clinical care accounts for about a fifth of variation in health outcomes [13-15].

There is an increasing call to leverage technology, specifically digital health technology (DHT), to support social needs and establish connections to resources and services. DHTs such as mobile health (mHealth) apps, telemedicine and telehealth services, health information technology (HIT) systems, and virtual reality (VR) and augmented reality (AR) technologies play increasing roles in the modern health care system. mHealth apps provide accessible platforms for health monitoring and management [16], telemedicine and telehealth offer remote health care access [17], HIT systems facilitate the integration of social needs data with medical care [18], and VR/AR technologies enhance training and patient engagement [19]. These technologies can systematically identify and tailor resources to individual needs, promoting personal health care management and reducing health disparities among low-SES populations [20]. Ideally, DHT should focus on increasing factors that promote health-related behaviors (eg, education) and decreasing factors discouraging health-related behaviors (eg, resource access barriers).

**Figure 1:**
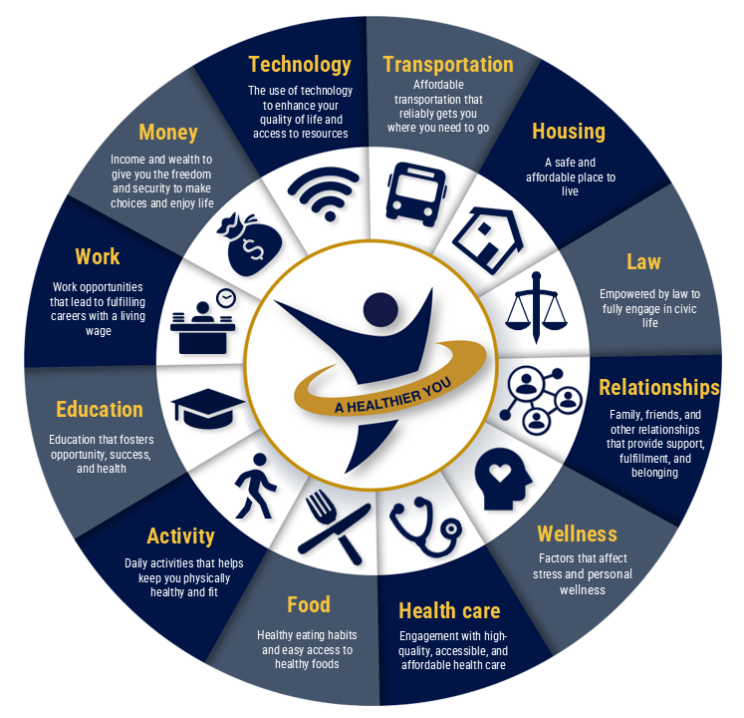
The 12 domains of social determinants of health (SDOH) used in this study.

A compelling case exists for targeting individuals of lower SES as SDOH-DHT primary users. This study focuses on SDOH-DHT among low-SES populations to capture the complex interplay of social, economic, and environmental factors influencing their health and health-related technology use. Although individuals of low SES have traditionally had limited access to digital technology [21], recent studies show a growing use of technology, particularly mobile phones, and mHealth apps among this population [8,22]. While the development of DHT apps has substantially increased, many do not meet the needs of people of low SES [8]. For example, engagement with digital health systems generally requires a basic level of digital health literacy, which is still in its early stages for many individuals of low SES [23]. Digital health literacy is “the ability to seek, find, understand, and appraise health information from electronic sources and apply the knowledge gained to addressing or solving a health problem [24].” Moreover, there is not currently a universal framework for assessing the effectiveness and usability of DHT in this population [8]. Furthermore, many SDOH-DHT apps are designed to address a single domain on SDOH, mostly commonly food and housing, but individuals of low SES must often address multiple SDOH domains that fluctuate throughout their lives [25] (Figure 1).

### Objectives

The manuscript aims to identify the characteristics, needs, and preferences of individuals with low SES using DHT to address HRSN. For digital SDOH management tools to best serve individuals of lower SES, we must use a user-centered design (UCD) approach that incorporates characteristics specific to the target user population, including contextual situations, preferences, and capabilities, to develop system requirements, goals, and design features [26,27]. In technology development, developers may be unfamiliar with the target user group, especially when specific user groups have historically been excluded from the design process [27,28]. UCD aims to shift developers’ focus from their own mental models to those of the targeted users [28]. This allows for a more user-empathetic, inclusive, and flexible approach to DHT development so that the functions, features, and aesthetics of SDOH-DHT meet the target user group’s needs [28].

Few studies have leveraged UCD to inform DHT addressing social needs among low-SES populations. In response to this gap, the first phase of this study uses UCD methods (eg, interview and focus group) and tools (eg, user profile) to address the following questions:

1. What are the DHT characteristics of SDOH-DHT users of low SES?

2. What are the needs and preferences of SDOH-DHT users of low SES?

The application of insights gained from exploring the characteristics, needs, and preferences of SDOH-DHT-targeted users raises the question of whether the current SDOH-DHT tools meet users “where they are.” Thus, we explore the following research question in the second phase of the study:

3. What identified needs and preferences do current SDOH-DHT address?

We then discuss how our findings can be applied to developing SDOH apps, particularly referencing the Florida International University (FIU) Thrive app currently being developed.

Overall, this study promotes an enhanced understanding of the target user population, desirable SDOH-DHT features and functions, and the status of existing SDOH-DHT in addressing those features and functions, and a discussion, with illustration, of how to collectively address desirable features with some focus on addressing differences between identified and current features and functions. As a result, findings and insights pave a pathway to advance SDOH-DHT design science and DHT product design.

## Methods

### Study Design

**Figure 2:**
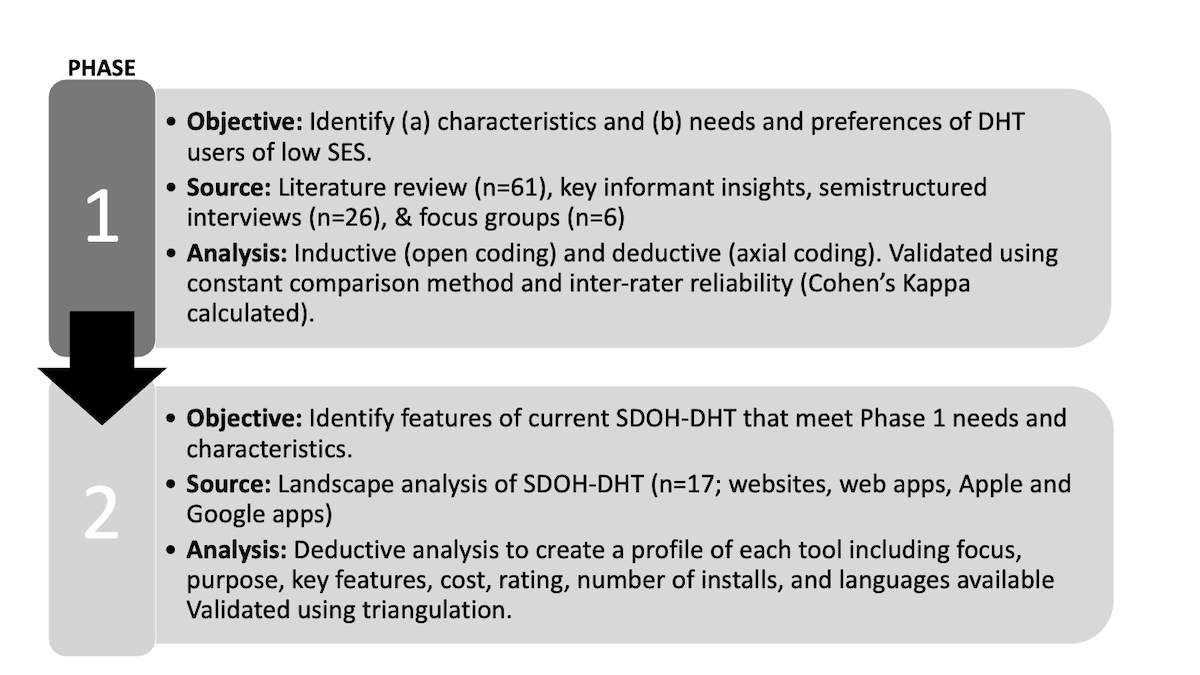
Mixed method approach including objective, data source, and analysis for each phase. DHT: digital health technology; SDOH: social determinants of health; SES: socioeconomic status.

We used a multiphase, mixed method UCD approach following the consolidated criteria for reporting qualitative research (COREQ) [29] to address our research questions (Figure 2 depicts an illustration of the methods). UCD seeks to incorporate all relevant stakeholders in developing system requirements, goals, and design features [26].

### Ethical Considerations

As this research involved human participants, ethics approval was obtained from the Institutional Review Board at FIU (IRB-22-0206). After the screening process for the interviews and focus groups, participants received an information sheet detailing the study procedures, risks, and benefits. At the beginning of each interview, the interviewer explained the study procedures, reaffirmed verbal consent for participation, and invited questions from participants. Participants were informed that they were free to withdraw from the study at any time. The data obtained from participants have been anonymized and are not identifiable. Following their participation, interview participants received compensation of US $25 in the form of a gift card.

### Phase 1: Understanding Users and Their Needs

Phase 1 occurred in the context of FIU Thrive, a trans-disciplinary design science research (DSR) study to use mHealth to address social risks and resulting disparities in vulnerable populations. DSR is a problem-solving approach that creates novel technologies and associated knowledge [30-32]. In this study, we report on our UCD efforts, which are integral to the more extensive FIU Thrive DSR study.

Phase 1 addressed our first 2 research questions on the characteristics, preferences, and needs among individuals of low SES for SDOH-DHT. Leveraging the expertise of UCD experts on our team and digital health literacy models (eg, Digital Literacies Domains and Relationships [33], ICT Digital Framework [34], and A Review of Digital Literacy Assessment Instruments [35]), we developed user profiles describing our target users based on their characteristics, prior experiences, and anticipated behavior to guide design choices [36]. The user profile was organized based on demographics, health/SDOH-related characteristics, and DHT-related characteristics of interest for this study and the overall FIU Thrive project (Multimedia Appendix 1 [8,35,37-77] shows categorized characteristics).

As a first step to completing the user profile framework, we conducted a focused narrative literature review to obtain generalized insight into the characteristics of interest for our target user population. Using multiple interdisciplinary databases (eg, Web of Science and PubMed), our search parameters combined low-SES terms with targeted characteristics or characteristic types aligning with our SDOH domains (eg, technology, wellness, health care, and health education; Figure 1) or SDOH terms (eg, social risk and social need). We performed a full-text review of each study to ensure relevance to our profile characteristics of interest.

To add detail to our user profile, we used aggregated data (eg, demographics and some social and health characteristics) from the records of The Green Family Foundation Neighborhood Health Education Learning Program (NeighborhoodHELP), a university-led community program providing interprofessional household-centered care for individuals who face challenges addressing social needs and are open to assistance [78,79]. These program members were targeted as the initial FIU Thrive pilot users. Our study population closely aligned with the literature’s definition of low SES: individuals or groups with limited income, education, and occupational prestige, often resulting in reduced access to resources and opportunities. NeighborhoodHELP aims to address social needs and maintain community connections, allowing us to address current and past priority needs with our participants. We aimed to leverage the firsthand experiences, needs, and preferences of individuals enrolled in the program to develop SDOH-DHT that effectively addresses their needs.

Guided by findings from our literature review and aggregate data, we tailored the development of target user interview and outreach worker focus group protocols to areas needing deeper exploration and gaps to be explored.

We constructed a semistructured interview protocol to explore SDOH-DHT user needs and preferences, adding depth and breadth to the user profile. We recognized three key aspects in developing a phenomenological interview guide: (1) contextualization, asking general questions related to participants’ general SDOH/social needs experiences and their management of these concerns; (2) apprehending the phenomenon, asking questions focused on participants’ experiences with using DHT to manage health and wellness concerns; and (3) clarifying the phenomenon, exploring areas for improvement in DHT for social concern management.

We developed a parallel structure for focus groups with targeted co-users, primarily NeighborhoodHELP outreach staff. The focus group protocols emphasized the attributes and deficits of currently used tools, the information needed to perform their jobs effectively, and their perception of the digital health capabilities of those they served.

The resulting interview and focus group protocols were reviewed and refined by a multidisciplined team involved with the Thrive project. This team included a researcher with substantial qualitative methods expertise (focused on information systems and health informatics) and familiarity with the study constructs, a primary care medical provider engaged in community outreach, a sociotechnical community health researcher experienced in developing technological platforms, and several team members with experience focused on UCD. A consensus was reached on the final protocols.

Participants (n=26, ages 20-71 years, mean age 50 years) were selected through purposive sampling to ensure representation across age decades within the lower SES demographic. The decision to include a diverse age range was guided by the recognition that age can significantly impact one’s perspective on socioeconomic challenges and resilience strategies [80,81]. By incorporating voices from younger and older individuals, we aimed to capture a broader spectrum of experiences and insights related to lower SES. NeighborhoodHELP outreach staff were trained in the purposes of the study and recruited program members to participate in the interview process, verifying that age (between 18 and 75 years) and capability (basic technical skills and English comprehension) inclusion criteria were met. For those who had difficulty with technology, the outreach specialist provided support to access Zoom. Participants whose primary language was Spanish or Haitian Creole were included if they could speak enough English to complete the pre-enrollment questions in English. Before each interview, recruiters obtained information from qualified and consented participants via telephone to characterize the nature of participant information technology (IT) use questions. This preliminary information provided insight and helped set the appropriate starting point for interview inquiries regarding technology.

We conducted 26 one-hour semistructured interviews with individuals of low SES via Zoom to obtain the end user perspective. Sample size guidelines for qualitative research suggest that having at least 20 participants is sufficient for data saturation, where incremental learning is minimal [82,83]. Interviews were conducted by 2 trained team members, one serving as lead and the other as scribe. The scribe could interject clarifying questions periodically during the interview to facilitate a comprehensive understanding. The scribe also provided a summary of interview highlights at the end of each interview, inviting participant comments regarding accuracy, omissions, and further explanation (ie, member check) [84].

For the focus groups, we recruited NeighborhoodHELP care providers aged 18 to 75, including clinical, outreach, administrative, faculty, and student divisions. These individuals have experience using a variety of electronic medical records (EMRs) and IT systems, including the university’s homegrown outreach health risk profile and outreach portal. Their familiarity with the challenges of collecting, maintaining, and reporting social risks, needs, goals, and patient-reported outcomes provided valuable insight into developing technological solutions. As with the focus groups, 2 research team members conducted the interviews as lead and scribe, performing a collective member check.

We conducted 6 confidential focus groups with a total of 28 participants. Typically, 3 to 5 focus groups per project are sufficient for achieving saturation [85]. We reached saturation by the fifth focus group, but conducted a sixth to ensure data saturation. Participants included physicians, outreach specialists, program coordinators, and social workers with an average of 3.8 years of experience working in the program. Most participants had prior experience working with underserved populations through other programs.

### Data Analysis

To enhance the reliability of the results, we performed member checks at the end of each interview and focus group by sharing interview summaries and highlights [84]. During these checks, we presented participants with highlights of our initial data analysis for feedback on accuracy and resonance with their experiences [86]. Deidentified interview and focus group transcripts were uploaded to Dedoose (Sociocultural Research Consultants) qualitative analysis software.

We used a hybrid thematic analysis approach using inductive and deductive coding [87]. Following guidance from King [88], we developed an a priori coding schema consisting of high-level concepts (ie, categories of the user profile and functional categories of the planned FIU Thrive app) for deductive coding (eg, member characteristics, areas of need -risk factors; SDOH data intake survey; app mock-up reactions; promoting app awareness, and implementation of app). These high-level concepts were applied to conceptually meaningful sections of the transcripts (aka slips) [89]. For instance, if participants discussed their perception of the Thrive DHT mock-up screen, we used app mock-up reactions.

Next, we further analyzed the selected slips of transcript texts coded at the deductive level using an inductive, open-coding approach to provide a more detailed and specific understanding of each high-level concept [90] (eg, details of technology use and reactions to screen mock-up). Specifically, we re-examined relevant portions of the text previously coded deductively under each high-level concept to create more discreet, detailed subcodes needed to provide a depth of insight into our first 2 research questions through an open coding approach.

Finally, we used axial coding to organize the discrete codes related to our first 2 research questions (user characteristics and SDOH-DHT needs and preferences) into broader, generalizable categories of the core themes presented in our results section [91].

We aimed to capture the rich, diverse narratives of participants and ideas as a collective set of data without subordinating or elevating the importance of any particular narrative or group of narratives (narratives belonging to a particular demographic group), providing valuable insights that can inform policy and practice aimed at addressing the challenges faced by this subpopulation. Therefore, while we may have had more individuals in a certain age group than others (or other demographic bin), data from each individual transcript was considered towards building an aggregated data set.

To ensure the trustworthiness of thematic analysis (ie, internal validity and reliability), we used Guba’s [84] strategies and Weber’s [92] recommendations for conducting a rigorous qualitative research study. These include peer debriefing (ie, code review), the code-recode procedure, and researcher triangulation. Table 1 summarizes these procedures.

**Table 1:** Summary of validity and reliability procedures.

Trustworthiness criteria and procedures		Description
**Internal validity**		
	Peer debriefing (code review)	A health communication expert reviewed high-level codes, their contextualized definitions, alignment between high-level codes and subcodes, the conceptual integrity of the coding schema, and 20% of data.
**Reliability**		
	Reproducibility	A graduate assistant (not involved in data collection/analysis) coded 15% of the excerpts using high-level coding schema. Cohen κ value was 0.99, indicating a high level of agreement.
	Stability	The lead researcher recoded 15% of data from the first 3 interviews approximately a month after coding. Cohen κ was 0.99, indicating a high level of coding stability.

### Phase 2: Landscape Analysis

Phase 2 involved a landscape analysis that included a structured review of available SDOH management technologies to identify whether their features aligned with the needs and preferences of health consumers, as identified in phase 1. This analysis did not involve direct usability testing, but rather examined publicly available information (eg, product descriptions and user documentation) to infer alignment with the needs and preferences identified by our target population. This approach is consistent with UCD methodologies that emphasize understanding the current design landscape from the mental model of users to inform new solution development [93]. For example, a recent study on diabetes self-management tools used a similar strategy to map available features against user needs as part of a UCD process [94]. Notably, as part of UCD, landscape analyses are critical to understanding the existing market of DHTs and identifying missing features and design elements that should be incorporated into new DHT designs [93-95].

To understand the current environment for SDOH apps and tools, we searched for website tools and web apps, as well as the Google Play and Apple App Stores using the search criteria presented in Table 2.

**Table 2:** Social determinants of health digital health technology (SDOH-DHT) search strategy.

	Web-based tools	App
Search Terms	One or more of these SDOH term permutations: Social determinants of health Social services Social services referral system SDOH Social Risks Social factors Social risk factors Social determinants Health‐related social needs Health‐related social problems Social needs Social needs-informed care Social needs-targeted care **AND** One or more of these digital term permutations: Digital health intervention Digital health technology Client communication system Community-based information system Digital health tools Interactive tools Tool Technology interventions E-health Website App Mobile health/mhealth Platform Connect	One or more of these SDOH term permutations: Social determinants of health Social services Social services referral system SDOH Social Risks Social factors Social risk factors Social determinants Health‐related social needs Health‐related social problems Social needs Social needs-informed care Social needs-targeted care
Inclusion criteria	Tool addresses more than one SDOH domain. Primary target user includes those experiencing a social needs consumer directed Interactive human-computer interaction (HCI) features included to provide SDOH education and awareness, assess social needs, obtain services, or manage social needs.	Tool addresses more than one SDOH domain. Primary target user includes those experiencing a social needs consumer directed Interactive-(HCI) features included to assess needs, obtain services, or manage needs.
Exclusion criteria	Tools that limit scope to one SDOH domain (eg, just health care needs). Tools that target primarily policy makers, community leaders, and health care providers or administrators (eg, tools that provide data sets and strategies for community health improvement)	Tools that limit scope to one SDOH domain (eg, just health care needs). Tools that target primarily policy makers, community leaders, and health care providers or administrators (eg, tools that provide data sets and strategies for community health improvement)

We followed several steps to identify the available SDOH technology tools targeting people with social needs. For website tools and resources, we performed an internet search using permutations of “SDOH technology tools” as a search term (Textbox 1 contains a complete listing). Search terms were identified through team discussion and literature search of relevant terms (eg, SDOH lexicon work by Alderwick and Gottlieb [2]). We included websites that included an interactive SDOH feature or specific training or guidance on implementing or evaluating SDOH-based programs. Websites that only provided general education about SDOH without targeted tools were excluded.

Next, we searched for smartphone tools and resources on the Google Play Store and the App Store using permutations of SDOH search terms (Table 2 contains a complete listing). We also reviewed apps suggested on the pages of other apps. Additionally, if we found an app on one platform (eg, Google Play Store), we searched for the same app on the alternative store (eg, App Store). We included any app that assisted professionals and community organizations in providing education, raising awareness, or alleviating more than one domain for people facing social challenges.

**Summary of social determinants of health digital health technology (SDOH-DHT) included in landscape analysis.**

**Mobile app (Apple):**

- Providers: EBT, debit, & more (Propel, Inc)
- What I Need (WIN) (OurCommunityLA)

**Mobile app (Apple and Android):**

- myCOMPASS PA (Pennsylvania State Government)

**Website and mobile app (Apple and Android):**

- FindHelp.org (FindHelo)
- HelpSteps (Boston Children’s Hospital)
- Your Texas Benefits (TX Health and Human Services Commission)
- ebtEDGE (Fidelity National Information Services, Inc)

**Website:**

- ACCESS Florida (Florida Department of Children and Families)
- Washington Connection - Your link to services (Washington Connection)
- Washington Healthplanfinder (Washington Healthplanfinder)
- 211.org (United Way Worldwide)
- Benefit Finder (US Government)
- HHS.gov Social Services (US Department of Health and Human Services)

**Web app:**

- ACT.md (Activate Care)
- NowPow (As of July 2024, the NowPow website states that the platform is no longer supported and available.) (Unite Us)
- Pieces (Pieces Technologies)
- WellSky Social Care Coordination

Three rounds of the search process were performed during the study to account for the inclusion of recently released tools in the final results. Two authors (CL and PD) conducted the final searches and data extraction during October 2024. It is of note that many tools were excluded as they only covered one domain. Indeed, we discovered that most existing apps and websites designed for at-risk individuals connect users to social services that address only a narrow range of social issues, leading those with more than one social need to choose to use technology tools to rely on multiple DHTs to meet their needs. The search process resulted in analyzing 17 DHTs (Textbox 1). Further details of these tools can be found in Multimedia Appendix 2.

We assessed each app based on the inclusion of clearly identifiable features from phase 1. In some cases, we were not able to gain full accessibility to some tools reviewed for inclusion due to privacy features (required a health provider registration and identification). In these instances, we consulted reference material on the tool features to help assure that we were not missing key features in our analysis.

## Results

### Overview

Our results provide insights into the characteristics of SDOH-DHT users and how currently available SDOH-DHT addresses their needs and preferences. First, we highlight insights regarding target user SDOH-DHT characteristics in detail in our user profile (Multimedia Appendix 1 [8,35,37-77]). Next, we review our findings regarding the needs and preferences of SDOH-DHT users and the associated landscape analysis results. This analysis is organized according to 5 themes: UCD; efficient solution-based assessment of SDOH risk/need; trust, privacy, and security; e-caring and support; and user feedback and education. To support the general themes and findings from the interviews and focus groups, we provide a detailed evidence trace table in Multimedia Appendix 3, moving from theme to associated open codes to representative quotes.

### Target User Characteristics

We defined our targeted profile based on the literature, focusing on individuals who experience a lack of resources such as food, education, income, and transportation, resulting in social risks and challenges. Phase 1 results revealed the DHT characteristics and needs of DHT users with low SES, particularly early adopters, allowing us to complete the comprehensive user profile in Multimedia Appendix 1 [8,35,37-77]. We augment this artifact with the highlights and extended descriptions provided below.

### Demographics

Our interview participants include individuals enrolled in NeighborhoodHELP to help address unmet social needs and risks. Table 3 contains the demographic information of our participants collected during screening.

**Table 3:** Interview participant demographic information.

Demographics		Values
Age (years), mean (range)		50 (20-71)
Household income, mean		US $18,519
Number of household members, mean		4
**Sex, n (%)**		
	Male	8 (31)
	Female	18 (69)
**Ethnicity, n (%)**		
	Hispanic or Latino	5 (19)
	Non-Hispanic or Latino	21 (81)
**Race, n (%)**		
	African American	18 (69)
	White	5 (19)
	Mixed race	2 (8)
	Asian	1 (4)

The age range of participants for our study was between 20 and 71 years, including representatives from each age decade to ensure a comprehensive understanding of how age influences the experiences of individuals within the low-SES population. The mean household income was US $18,519, and the average household size was 4 members. While English-speaking was required for interview participation, linguistic diversity is present within the overall NeighborhoodHELP population. Among the NeighborhoodHELP population, 40% of members primarily speak Spanish and 14% speak Haitian Creole. Educational attainment varied, with 77% having completed at least high school.

### The Social Needs Landscape: Insights From Participants and Professionals

Participants reported a wide array of social needs, reflecting the complex challenges low-SES populations face. These needs spanned financial instability, food insecurity, housing concerns, transportation difficulties, unemployment, educational barriers, limited health care access, and inadequate social support. Interviewees provided examples of how issues in the social domains, such as education, finances, and food insecurity, negatively impacted their physical and emotional health.

Participants indicated that they attempted to address their social needs through various resources, such as governmental programs, churches, and local nonprofits. When trying to address these needs, participants encountered several obstacles, including fragmented services, burdensome application processes, financial constraints, and competing life demands that made it challenging to navigate support systems effectively.

Participating professionals indicated that successful care plans addressing social needs must include a triad of individual patient priorities, the feasibility of meeting specific needs, and evidence-based guidelines. They also noted that identifying the target user’s strengths alongside risk areas is beneficial as these strengths can serve as leverage points and motivators.

### DHT Use

Studies show that individuals with low SES often struggle with IT literacy, making it difficult to adapt to new tools. However, our research reveals that during the COVID-19 pandemic, many reported improved digital health use. Focus group participants highlighted various programs that promoted the use of technology, particularly for completing applications and checking statuses. Despite low SES, participants demonstrated significant engagement with digital technologies. Most (81%, n=21) reported daily internet use, and 81% (n=21) regularly used smartphone apps. However, self-assessed digital skills varied widely among participants, indicating a range of comfort levels with technology. Still, about half of the study participants used DHT for various health-related social activities, including scheduling health care appointments, completing social need applications, reading health care reviews, monitoring symptoms, accessing EMRs, retrieving medical records, completing health surveys, and using health-related apps (Multimedia Appendix 4).

### User Needs, Preferences, and Current Technology Solutions

Our user profile work and detailed qualitative analysis resulted in 5 key themes related to user needs and preferences: UCD, solution-based assessment of social needs, trust, privacy and security, e-caring and support, and education and user feedback. The following sections explore these themes identified through qualitative analysis of target user needs and preference data and share the presence of features associated with these themes in current DHT offerings. Table 4 highlights key comparisons from our landscape analysis, showing how current DHT aligns with or diverges from the identified needs and preferences. This comparative narrative addresses our research question on how the current landscape meets—or fails to meet—these needs. Multimedia Appendix 2 includes detailed descriptions of each SDOH-DHT we explored for a cohesive depiction of the landscape.

**Table 4:** Overview of themes from literature review, interviews, focus groups, and landscape analysis.

User needs and preferences		Current DHT^a^ identified
**User-centered design**		
	Multilingual	Few offer language translation beyond Spanish (eg, Creole)
	Use of images, icons, and videos over text to help address literacy and add to aesthetics	Limited use of images and videos; most DHTs are text-based with few illustrations
	Customized needs prioritization, goal setting, messaging (eg, reminders and progress tracking with incentivization/encouraging messages)	No DHTs found that allow easy assessment and prioritization of needs and goals; users often cannot choose notification timing
	Self-paced, streamlined survey completion process that meets users where they are and their schedules	DHTs break questions into sections but do not allow users to save progress; few offer prefilled responses to streamline processes
**Efficient,** **solution-based assessment of social risk/need**		
	One-stop access to resources addressing multiple social domains; cross-domain referencing	No DHTs surveyed include guidance to assess and navigate all health-related social domains with direct access by users; users need multiple DHTs, specific ideas about what services they need, or a case manager to assess needs and address barriers
	Multiple referrals filtered by goals, geography, open hours, eligibility, etc.	Resources are searchable by keyword or domain area; some offer zip code filtering
	Track progress over time with outreach workers/administrators	Program-focused DHTs track eligibility, application progress, and benefits status but are limited to those receiving formal assistance
	Easy sharing of data with health care providers/social service professionals	Patient SDOH status integration into other systems (eg, EMRs) is generally unavailable for patient use
**E-caring and support**		
	Use of empathetic and cooperative tone and language; motivational messaging	Few DHTs show evidence of an empathetic tone or motivational messaging
	Option for continued contact with outreach workers; surrogate users (household, friends, outreach workers)	Several DHTs are designed for health care providers, not the general public; some offer training/resources for addressing social needs, while others only provide search functionality for services
**User education and feedback**		
	DHT skill training (eg, swipe function) and health information searching; education on taking action on social needs	Few DHTs offer education on taking action to address social needs or risks; existing training is often limited to basic use of DHT or provider training
	Review existing feedback (reviews and ratings) and add personal feedback	Most SDOH-DHT apps have user reviews in app stores; few include feedback from users and outreach workers on past service experiences
**Trust, privacy, and security**		
	Trustworthy DHT platform and sponsor; evidence of safeguards against hackers, scams, and spam	Few current SDOH-DHTs prominently display privacy and data security policies or trust language
	Information is optional, particularly for sensitive topics, unless required for app processes	Many DHTs do not allow users to skip sensitive questions

^a^DHT: digital health technology.

### User-Centered Design

The UCD process tailors app design and development to the needs and capabilities of the target user population. Our data highlighted 3 key areas of UCD needs and preferences: inclusiveness, customization, and human-computer interaction efficiency. Although some existing apps addressed 1 or 2 of these areas, most apps in our landscape analysis lacked comprehensive attention to all aspects of UCD.

### Inclusiveness

Participants strongly emphasized the need for inclusive design in SDOH-DHT. They expressed a desire for multilingual options, particularly highlighting the importance of Spanish and Haitian Creole translations alongside English. This linguistic inclusivity was seen as crucial for ensuring that diverse user groups could effectively engage with the technology. Our participants preferred apps in English, Spanish, and Creole. While several DHTs offer Spanish translations (eg, Your Texas Benefit, ebtEDGE, myCOMPASS PA), very few include Creole (eg, ACCESS Florida).

Additionally, participants stressed the importance of using simple, accessible language to accommodate varying literacy levels within the target population. Participants also noted that simpler terminology could reduce on-screen text and suggested that icons, graphics, and videos could further enhance usability. Some participants used sites like WebMD for their illustrative content, but few SDOH-DHTs include such features. For instance, ACCESS Florida (Florida Department of Children and Families) offers limited visuals, and while ACT.md (Activate Care) and WellSky Social Care Coordination (Wellsky) provide videos, these are general overviews rather than guides for accessing specific services. Most DHTs in our analysis were text-heavy, offering questionnaires or service lists with minimal visual aids (eg, Benefit Finder).

### Customization

Customization emerged as another critical aspect of UCD. Participants wanted the ability to prioritize their needs within the app, set personalized goals, and receive customized messaging. Current SDOH-DHTs for individual use often fail to account for personal factors and prioritized needs.

Participants envisioned features such as reminders and progress tracking accompanied by encouraging messages, which they felt would enhance their engagement and motivation to address their social needs. While many existing SDOH-DHTs, especially mobile apps, offer notifications, they often lack options for users to choose when notifications should appear. For example, the “Providers: EBT, debit, & more” app (Propel, Inc) allows users to receive notifications for benefits, offers, tax filing, and promotions, but does not let them customize the frequency of these alerts.

### Efficiency

Efficiency in interactions was also a key concern. Participants expressed a preference for a self-paced, streamlined survey completion process. They indicated that ideally, survey sessions should be limited to 10-30 questions and last no more than 20 minutes. This preference reflects the time constraints and competing priorities many individuals of low SES face. However, some SDOH-DHTs, like the Benefit Finder (US government), require up to 30 minutes to complete a questionnaire, which does not align with these preferences. While about one-third of current SDOH-DHTs avoid repetitive and lengthy data collection, some still take up to 30 minutes to complete.

Furthermore, participants stressed the importance of completing surveys at their own pace, starting with the most critical sections and saving their progress to continue later. The ability to save progress helps avoid repeating processes, which participants found essential. However, most web-based DHTs do not allow users to save their progress. For example, ACCESS Florida (Florida Department of Children and Families), Benefit Finder (US Government), and Washington Connection require users to fill out entire questionnaires without the option to save and return later. While some DHTs, like ACCESS Florida (Florida Department of Children and Families), break down questions by sections (eg, household, income, expenses), they still do not allow users to save partially completed questionnaires.

### Efficient Solution-Based Assessment of Health-Related Social Risks, Needs, and Goals

Users articulated a strong desire for comprehensive tools integrated with practical solutions. They envisioned a “one-stop” platform that could address multiple social domains simultaneously, recognizing the interconnected nature of many social needs. For example, one participant faced food insecurity but could not access a food pantry due to a lack of transportation. Others were ineligible for services because of their immigration status. Participants expressed frustration with fragmented services and saw great value in cross-domain referencing within a single platform. Tools with a broader scope of social needs typically cover housing, food, employment, health care, transportation, and childcare services. For example, FindHelp (FindHelp) spans domains including food, housing, transportation, health, money, education, work, and legal services.

Social needs navigation professionals also emphasized the importance of tracking progress in addressing specific social needs. However, current DHTs typically do not integrate social needs assessments with service referrals or track progress over time. Some platforms, like Pieces (Pieces Technologies), use artificial intelligence and advanced analytics to automate screening and address SDOH issues through predictive modeling of at-risk patients in EMR systems. Unfortunately, these advanced algorithms and processes are inaccessible to the general low-SES population.

Another key preference was the ability to receive filtered referrals based on specific criteria. Users wanted to be able to narrow down service options based on their personal goals, geographic location, service hours, and eligibility criteria. This level of customization was seen as essential for making the platform truly useful in addressing individual needs. Most SDOH-DHTs provide lists of service referrals for specific needs and guide users to access a single service, but do not automatically filter resources based on user eligibility and practical considerations like access. More comprehensive tools, such as HelpSteps (Boston Children’s Hospital) in Massachusetts and FindHelp (FindHelp) nationally, serve as central resources for individuals but are primarily designed for resource searching rather than automatically filtering resources based on eligibility. These tools allow users to search for help by directly finding and connecting with services like food, housing, health care, and other types of support available nearby, usually by entering a zip code.

Participants also emphasized the importance of easy data sharing with health care providers and social service professionals. They recognized the potential for improved care coordination if their social needs data could be seamlessly integrated with their medical care. Administrators and outreach workers suggested that integrating this functionality with EMR systems could be an effective solution. While some DHT platforms are already integrated with EMRs, these are typically designed to assist providers rather than directly support low-SES patients. Certain platforms, such as NowPow (Unite Us) and WellSky Social Care Coordination (WellSky), focus on building networks among community care providers.

### E-Caring and Support

The concept of “e-caring” also emerged as a significant theme in our discussions. Participants valued an empathetic and cooperative tone in all interactions within the app. They expressed a desire for motivational messaging, particularly when completing tasks or reaching milestones, as a way to maintain engagement and boost morale. However, our landscape analysis revealed that few existing DHTs offer motivational messaging when users complete questionnaires or select different services.

Many participants also highlighted the importance of having options for continued contact with outreach workers or support from surrogate users, such as household members or friends. This human element was seen as a crucial complement to the digital platform, providing personalized support and guidance when needed. During our landscape analysis, we identified ACT.md (Activate Care), an app that integrates with EMRs. The ACT.md platform enables health care providers to manage patient needs and coordinate care with community partners by sharing tasks, messages, and data across organizations. Care teams can view patients’ unmet social needs and collaborate with community organizations to address those needs. However, these platforms are only accessible to providers and patients within health care systems that have purchased them. Additionally, they are primarily designed for health care providers rather than individual users.

### User Education and Feedback

Participants showed interest in educational content within the SDOH-DHT, but with a specific focus on actionable information. Rather than basic facts about SDOH, they wanted practical guidance on how to address their specific social needs. Practical guidance references included a desire for DHT skill training (such as instruction on navigation functions) and advice on how to search for health information effectively. Existing digital health technologies tend to focus on offering general information across various social domains (eg, Your Texas Benefits, HHS.gov Social Services) or screening users for eligibility for certain benefits (eg, Benefit Finder*,* Washington Connection - Your Link to Services) without providing step-by-step guidance on how to address the identified needs and risks effectively.

The ability to engage with feedback was another important aspect for users. Participants wanted to be able to review existing feedback, including ratings and reviews of services, as well as contribute their feedback based on their experiences. This 2-way flow of information was seen as valuable for making informed decisions about services and contributing to community knowledge. While most SDOH-DHT apps feature user reviews on the technology itself in the app store, only 2 apps included feedback from both users and outreach workers on past service experiences. For instance, ACT.md, a platform that enables health care providers to manage patient needs and coordinate care with community partners, includes a page dedicated to client experiences. This page details how the platform has assisted its clients and features quotes from clients about their experiences with the programs.

### Trust, Privacy, and Security

Trust, privacy, and security emerged as foundational concerns for potential SDOH-DHT users. Participants emphasized the need for clear evidence of safeguards against possible threats such as hackers, scams, and spam. They were particularly sensitive to the possibility of personal information misuse leading to unwanted solicitations. Our landscape analysis showed that only a few current SDOH-DHTs prominently feature clear privacy and data security policies. One exception is the Pieces DHT, which offers users a dedicated page outlining its data privacy policy and contact information for any questions or concerns.

Regarding data sharing, participants strongly preferred granular control over their information. They wanted the option to selectively disclose sensitive information, with mandatory fields limited to only what was absolutely necessary for specific processes. Existing DHTs, especially those using questionnaires, often do not allow users to skip these sensitive questions. For example, the Benefit Finder (US government) requires users to disclose citizenship status, annual income, and employment status before allowing them to proceed with selecting services. This mandatory disclosure can deter some users from using the platform. Transparent privacy policies were seen as essential for building trust in the platform.

## Discussion

### Principal Findings

Our study reveals the characteristics, needs, and preferences of individuals of low SES using SDOH-DHT and how current DHTs align with or diverge from these needs and preferences. Our results, identified across the key themes such as UCD, solution-based features, e-caring support, education and feedback, and trust and security, have important implications for the effectiveness, adoption, and equity of digital health interventions targeting SDOH. The pervasive message users have communicated is the need for continuity across all themes.

**Figure 3:**
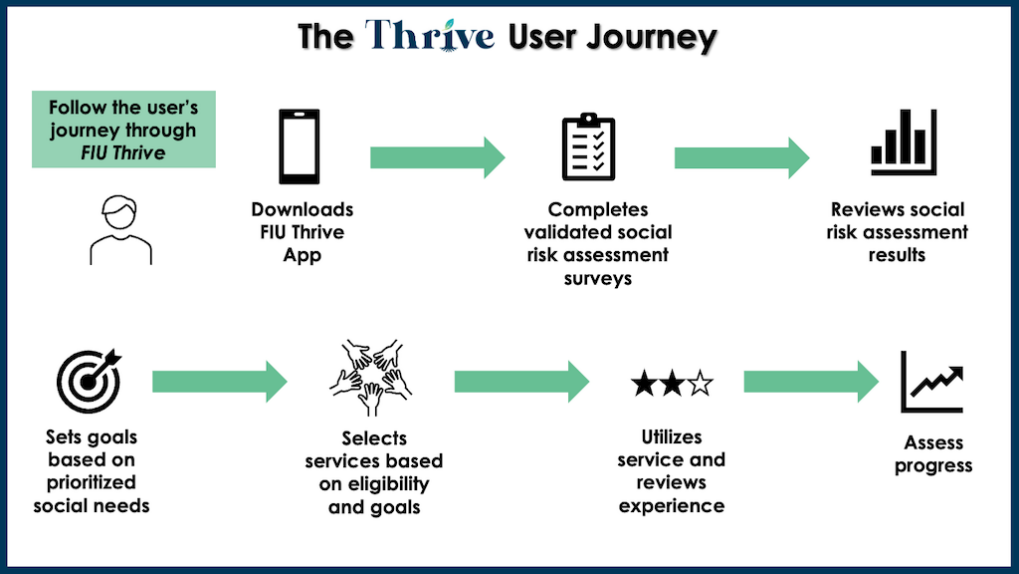
Thrive user journey. FIU: Florida International University.

The insights gained from this study are crucial for informing the iterative design process of the FIU Thrive mHealth tool. FIU Thrive aims to maintain continuity in addressing social needs by creating the user journey. This mHealth tool uniquely emphasizes identifying and addressing users’ health risks, needs, priorities, and goals. It integrates these elements into actionable insights, creating individualized care plans that enhance self-knowledge and empower individuals. Users can connect with recommended services based on their specified social risks, goals, and geographic area. FIU Thrive aims to address risks and needs across multiple SDOH domains. Overall, the FIU Thrive user will embark on a journey to understand their social needs and risks better and find evidence-based and personalized solutions to address them. The FIU Thrive User Journey is presented in Figure 3.

FIU Thrive also seeks to address the needs and preferences of users of low SES within the different themes. Based on this UCD study, features designed into FIU Thrive to address the needs and preferences of our target users are presented in Textbox 2 and further described below.

The gaps in current SDOH-DHT may perpetuate barriers to access and engagement for individuals of low SES, who often face language, literacy, and cultural challenges [8,23,24,26-28]. Features for improving aspects of UCD to tailor app design and development to the needs and capabilities of the intended user population include multilingual support, use of graphics for visual guidance, customization, and self-paced and streamlined data collection. By interviewing participants aged 20-71 years, we captured a broad spectrum of experiences and insights related to lower SES. Decreased DHT adoption can be attributed to biopsychosocial factors that can be generalized to many age groups, including cognitive and physical impairments, lack of familiarity with technology, or lack of device access [96,97]. Based on current literature and our results, strategies to accommodate these needs include technology features such as larger font size and instructional videos, voice-to-text functions, and increased support from family, caregivers, or health care providers [96,97].

The fragmented nature of current SDOH-DHT resources, coupled with limited integration of referral and tracking features, may impose additional burdens on individuals of low SES who often navigate complex and disconnected social service systems [5,98]. The absence of comprehensive, coordinated support can lead to unmet social needs, delays in accessing care, and poorer health outcomes [3,27,99]. Improving solution-based assessment of HRSN by offering integrated, one-stop access to a wide range of social services, personalized referrals, eligibility screening for social services, and progress monitoring (for managing social risks) could help streamline connecting users to appropriate resources and support [6,20,100].

The lack of empathetic support and action-oriented education in existing tools may also undermine user engagement and motivation, particularly among individuals facing multiple stressors, competing priorities, and limited social support [27-29,99]. While using DHT platforms, our users emphasized the importance of conveying sensitivity, empathy, and motivation, as positive in-person interactions with health care providers do. Participants reported that features conveying e-caring included using empathetic and cooperative language, motivational messaging, and web-based chat or support group features.

**Florida International University (FIU) Thrive app features.**

The FIU Thrive mHealth app is designed to address the specific needs and preferences of low socioeconomic status users identified in this study. The app is designed to incorporate the following features:

1. Multilingual support (user-centered design [UCD])

- In-app language selection option (eg, English, Spanish, and Haitian Creole)
- Automatic language detection based on the user’s device settings

2. Simple and intuitive user interface (UCD)

- Clean, uncluttered design with minimal text
- Use of icons and visual cues to guide navigation

3. Customized user experience (UCD)

- Customizable user profile
- Ability to set personal goals and priorities
- Tailored content and recommendations based on user’s social risks/needs assessment results

4. Solution-based assessment of social risk/need

- Brief, user-friendly questionnaires for each social domain
- Progress tracking and visualization using color-coded SDOH wheel
- Option to complete assessments in multiple sessions

5. Integrated referral system

- Personalized referrals to local resources and services based on social needs assessment results
- Detailed information about each referral, including eligibility criteria, contact information, and user ratings
- Ability to save and track referrals within the app

6. E-caring support features

- In-app messaging with outreach workers and peer support groups
- Encouraging notifications and reminders to support goal progress
- Web-based coaching and educational content tailored to user’s needs

7. User education and feedback mechanisms

- User feedback on referral services
- In-app feedback form to report issues or suggest improvements
- Regular prompts to share progress and successes with outreach workers

8. Robust privacy and security measures

- Secure user authentication and data encryption
- Granular privacy settings to control data sharing
- Clear, concise privacy policy and terms of service

Regarding references of e-caring to their current technology, commonly used apps manifesting some aspect of e-caring that impacted our participants’ health and wellness were spiritual and fitness apps. Participants reported continual use of spiritual and fitness apps because of the encouragement and motivation they received through notifications and messages, ensuring the user that they are moving in the right direction and imparting a sense of accomplishment. Participants reported that these messages helped them feel cared for and as if they received individualized attention to their actions, reducing the monotony of the DHT interaction. Ultimately, our participants appreciated that these messages allowed them to obtain reassurance and affirmation without physical interactions from a provider. Some participants commented that these elements of e-caring were an essential component of the long-term use of DHT. Our study supports that designing for e-caring and support (eg, “individualized attention”), which combines technology’s efficiency with human interaction’s empathy, may be particularly valuable in building trusting relationships and promoting sustained engagement [101].

Incorporating increased user education and feedback enhances engagement and impact of SDOH-DHT for this population [8,102,103]. For example, participants indicated that they desired the apps to enhance their knowledge and skills and provide useful feedback in the following areas: IT skill training, education on taking action to address HRSN, and a feedback/ rating mechanism for social services consulted.

Privacy and security concerns emerge as a critical barrier to trust and adoption of SDOH-DHT among users of low SES who may have heightened sensitivities around the protection of their personal information, given experiences of discrimination, stigma, or exploitation [104,105]. The absence of clear privacy policies, granular data-sharing controls, and secure communication channels in many current tools may exacerbate these concerns and deter engagement [106]. Developing SDOH-DHT with robust privacy safeguards, transparent data practices, and user control over information sharing is essential to fostering trust, buy-in, and long-term use among low-SES populations [107,108].

Our findings highlight the importance of addressing HRSN among low-SES populations through various DHTs. mHealth apps provide accessible health monitoring and management platforms, crucial for low-SES populations facing barriers to traditional health care [16]. Telemedicine and telehealth services offer remote health care access, addressing transportation barriers and improving care for underserved communities [17]. HIT systems facilitate the integration of social needs data with medical care, enabling providers to better address the social determinants affecting patients’ health [18]. EHR interoperability is crucial in achieving seamless communication and coordination of care. While VR and AR technologies are increasingly used in surgery [109] and occupational health to enhance training and patient engagement [19], their direct application to addressing HRSN among low-SES populations may be less clear. VR has been used in education related to SDOH to help providers recognize situations and increase empathy in management [110]. However, incorporating e-caring support features and personalized feedback mechanisms can improve the user experience and effectiveness of VR and AR applications for low SES populations [111].

In low and middle-income countries, barriers to DHT implementation include infrastructure limitations (unreliable electricity, limited internet), insufficient resources, and poor digital literacy among patients and providers [112,113]. Key facilitators include continuous on-the-job training, strong stakeholder commitment, and technologies designed with user-friendly interfaces [113]. Simpler DHTs such as telemedicine, mHealth, and EMR (including patient portal access or printouts) may be best initial DHT options in low and middle-income countries as they are easiest to implement due to lower infrastructure requirements, and easier integration into workflows [112]. Current literature has shown that efforts to bring DHT to such countries have been successful in improving the standard of health care service delivery, data management for decision-making, and boosting attendance at health facilities and use of services [112,113]. Therefore, we see promise in adapting our results in many settings, including low and middle-income countries, with adaptation as needed.

Overall, future work should focus on developing and evaluating SDOH-DHT that incorporates the user needs and preferences identified in this study. This could involve the creation of new tools (eg, FIU Thrive) or adapting existing platforms to better align with the requirements of those with low SES. Importantly, these development efforts require a solid commitment to UCD principles and the meaningful engagement of diverse individuals as co-designers and cocreators throughout the process to facilitate usability and acceptance. By involving target users as active partners in the conception, design, and iteration of SDOH-DHT, we can ensure that these tools are aligned with their unique needs, values, and lived experiences [37]. This participatory approach can help to identify and address potential barriers to adoption and use, enhance the cultural responsiveness and contextual relevance of the tools, and ultimately, improve their acceptance, usability, and impact among populations with low SES [114-116].

Furthermore, the development and implementation of SDOH-DHT must be situated within a broader ecosystem of supportive policies, programs, and partnerships that address the root causes of health disparities [3]. Digital tools alone cannot solve the complex, systemic issues that contribute to health disparities. Still, they can play a valuable role in connecting individuals to resources and facilitating communication and coordination among service providers [117].

### Limitations

The purposive sampling method, while effective for ensuring representation, may limit the generalizability of our findings to the broader population of lower SES individuals. Additionally, the qualitative nature of the study means that the findings are inherently subjective and may not reflect the experiences of all individuals within this demographic. Limitations of our study also include the focus on participants speaking at a threshold level of English (speaking enough English as a primary or secondary language to complete the pre-enrollment questions in English) and the potential for incomplete identification of relevant SDOH-DHT given sample limitations. While we aimed to include a diverse sample of target users, the lack of non-English speaking participants may limit the generalizability of our findings to populations with limited English proficiency. Future research should use a cross-sectional design study with a larger and more representative sample of North American populations, including First Nations individuals and African immigrants, to capture the unique needs and preferences of a diverse patient population.

Additionally, despite our systematic search strategy, it is possible that some relevant SDOH-DHT were not identified or included in our landscape analysis. Moreover, due to the proprietary nature of some of the systems, we were unable to access some parts of some systems and include functionality of these parts (beyond what was described and referenced in information materials) in our landscape analysis. As the field of digital health is rapidly evolving, new tools and platforms may have emerged since our data collection in May 2024. Ongoing research should continue to monitor and evaluate the SDOH-DHT landscape to ensure that the most current and relevant tools are considered.

### Conclusions

This study identifies characteristics, needs, and preferences related to SDOH-DHT among individuals of low SES compared with the current DHT landscape. Five themes emerged from our analysis: UCD, solution-based assessment of social risks and needs, e-caring and support, education and feedback, and trust, privacy, and security. This study also identified gaps between the needs and preferences of individuals of low SES for comprehensive social needs support and the features of current SDOH-DHT. Discrepancies between target user needs and current DHT features represent missed opportunities in developing user-centered tools to assist individuals with self-management of social needs. These findings underscore the need to design SDOH digital health interventions that are inclusive, empowering, and responsive to the unique challenges faced by these populations.

By creating digital health interventions that strive to truly meet the needs and preferences of users with low SES, we can harness the power of technology to bridge health disparities and promote public health. However, the development of user-centered SDOH-DHT is just one piece of the puzzle. We can only create the enabling environment needed for SDOH-DHT to achieve its full potential in advancing health and reducing health disparities by working together across disciplines, sectors, and communities.

## Appendix

Multimedia Appendix 1 User profile low-socioeconomic status individual as potential social determinants of health mobile health user.

Multimedia Appendix 2 Social determinants of health digital health technology landscape analysis.

Multimedia Appendix 3 Evidence trace table.

Multimedia Appendix 4 Digital health technology user characteristics from screening survey.

## Abbreviations

AR: augmented reality
COREQ: consolidated criteria for reporting qualitative research
DHT: digital health technology
DSR: design science research
EMR: electronic medical record
FIU: Florida International University
HIT: health information technology
HRSN: health-related social needs
IT: information technology
mHealth: mobile health
NeighborhoodHELP: Neighborhood Health Education Learning Program
SDOH: social determinants of health
SES: socioeconomic status
UCD: user-centered design
VR: virtual reality
