# Supplementary material for: Evaluating the Needs and Characteristics of Individuals of Low Socioeconomic Status Using Digital Health Technology to Address Health-Related Social Needs: Mixed Methods Study With Patients and Care Providers
Source: JMIR Hum Factors. 2025 Sep 12;12:e69545. doi: 10.2196/69545 (PMC12475883; doi:10.2196/69545)
Supplement: Multimedia Appendix 1 [file humanfactors_v12i1e69545_app1.docx]

# Appendix 1. User Profile – Low-SES individual as potential SDOH mHealth user

| Characteristics of Target Users with Low SES | Source of Data | | |
| --- | --- | --- | --- |
|  | Literature | NeighborhoodHELP leadership | Interviews & Focus Groups |
| Demographics |  |  |  |
| Low-SES Criteria | SES measured by resources (education, income), absolute poverty measures (185% Federal Poverty Line based on family size [1]), or relative poverty measures (food insecurity, income-to-needs ratio) | Uninsured individuals of low SES who have challenges addressing social needs appreciate having help managing their social needs. | Includes undocumented immigrants facing unique barriers. |
| Native Language |  | Preferred Language: Spanish (1074; 41.3%) English (1061; 40.8%) Creole (350; 13.5%) | English as a Second language speakers report difficulties understanding directions and questions on digital platforms. |
| Visual, Auditory, and Mobility Limitations in Technology Use | Physical restrictions, such as vision or manual dexterity declines, affect older adults' technology use. [2, Ch 6] | 73% of household members can perform daily activities without any limitations. | Accessibility issues often arise due to language, disability, or technological limitations. |
| Literacy/ Learning Style | Education level can influence literacy and learning preferences, with gaps in health and digital literacy.  [2, Ch. 3] | 77% of household members have a GED or higher | Users prefer simple, practical training for using DHTs and often seek assistance from family or others for support. |
| Health/SDOH | | | |
| Knowledge of Health/SDOH | - 9/10 adults may not possess the skills they need to manage their health and prevent disease. [3] - Low health literacy is associated with limited knowledge of SDOH [4] - Many Americans are unaware of the impact of SDOH on clinical outcomes [5] - There is often unawareness of the broader factors (e.g., discriminatory policies, environmental hazards) that constitute SDOH. [5] | Users are generally aware of their social needs but lack knowledge of available services and how to access them. | - Users experience the negative impact of SDOH on health and healthcare access. - Some users make connections between their social needs and physical or mental health when prompted, recognizing how one need can affect another.   • Challenges in addressing social needs are common, compounded by limitations in health and digital literacy, which make it difficult to understand available resources or services. |
| Most prevalent SDOH Needs | - Activity   - In 2020, adults in households with family income at or above 200% of the federal poverty line were more likely to meet both physical activity guidelines than those in lower-income households [6] - Education   - In 2016, young people aged 16 to 24 from low-income families were 3.7 times more likely to have not completed or been enrolled in high school compared to those from high-income families [7, 8] - Food   - In 2020, 28.6% of low-income households faced food insecurity [9, 10] - Healthcare   - In 2021, people in low-income households experienced worse access to care than those in high-income households for 71% of the measures included in the National Healthcare Quality and Disparities Report (NHQDR), such as health insurance coverage, having a usual source of care, facing fewer difficulties when seeking care, and receiving timely care [11] - Housing   - In 2019, 83.5% of households earning less than $15,000 a year were cost burdened (spend > 30% of their income on housing) [12, 13] - Law   - In 2022, individuals with family income <$50K made up 26% of voters and 52% of nonvoters in the 2022 midterm elections [14] - Money   - In 2020, the U.S. poverty rate was 11.4% (37.2 million people)[15, 16] - Relationships   - On average, individuals of individuals of above-median-SES have 25.4% more friends than individuals of below-median-SES [17] - Technology   - In 2021, 63% of adults in households with annual incomes of $100,000 or more had home broadband, a smartphone, a desktop or laptop computer, and a tablet, compared to 23% of those in lower-income households [18] - Transportation   - In 2022, 15.8% adults with family incomes less than 100% of the federal poverty level lacked reliable transportation for daily living compared to 2.9% for those with family incomes of 400% of the federal poverty level or greater [19] - Wellness   - From 2009 to 2013, 8.7% of adults with income below the federal poverty level reported serious psychological distress, while only 1.2% of adults earning 400% or more of the poverty level experienced similar distress [20] - Work   - In 2021, 6.4 million individuals were considered “working-poor” (spent ≥ 27 weeks in the labor force with incomes below the official poverty level) [21] | The SDOH domains assessed by the outreach team include income (with assistance in benefits application), education and life skills, legal (immigration), employment, healthcare access, food, housing, daily activities (including behavioral health and disability), technology (internet access), and transportation. | Users tend to focus on addressing their most immediate needs first, frequently excluding services that are unavailable or too far from their location. |
| General Attitude/Motivation towards Health/SDOH Management | - Lower health consciousness and a tendency to think less about the future [22] - Belief in chance as a strong determinant of health, leading to lower self-discipline [22] - Misunderstanding of upstream drivers of health due to misplaced focus on individual behaviors [5] - Adherence to health and SDOH management is often hindered by financial limitations, lack of information, and competing life commitments [23] - Some individuals seek to understand their full health/SDOH assessment, while others avoid it due to the fear of added stress [24] | Attitudes vary significantly depending on the individual's situation and characteristics. Some actively follow up on their health/SDOH needs, while others are less consistent in doing so. | - Users are generally open to participating in social risk assessments and screenings. - Social risk assessments are seen as useful in identifying areas of need. |
| Current Self-directed Practices in Managing SDOH | Health and social workers play a key role in facilitating collective goal setting for addressing various SDOH issues [25-27] | - Practices vary widely among users. Some are able to manage their social needs independently, while others rely heavily on outreach workers or face challenges in meeting basic needs. - Knowledge and use of public resources, such as FindHelp.org or individual agencies, also vary greatly. - Available resources range from public benefits to community-based, faith-based organizations, and other local agencies. | - Users express a desire for assistance in setting goals to address social needs. - Selection of resources and services is often based on feedback or reviews from others. - Many services are ruled out due to geographic limitations. |
| Challenges / Difficulties Regarding SDOH | - Lack of sustainable, scalable solutions for addressing SDOH [26, 28] - Life commitments often interfere with the ability to manage SDOH [23] - Low information awareness among users [23] | - Services are often fragmented, with varying eligibility and application requirements. - Users face challenges in understanding service availability, eligibility, and how to apply, along with transportation and legal barriers. - Satisfaction with services varies depending on the type of need, location of services, and eligibility challenges. | - Service lists are helpful but frequently ignored due to time constraints, perceived inapplicability, or geographic limitations. - Undocumented individuals often perceive most services as inaccessible. - Online searches are problematic, frequently resulting in distant or irrelevant services. - Users face issues such as unresponsive phone numbers, unlisted service hours, and reluctance to rate or review services. - Frustration is common with service quality, including lack of follow-up and receiving outdated food from food banks. |
| How have their SDOH needs/services provided changed due to COVID? | - Increased technology use for remote work and education [29] - COVID infection is associated with economic disadvantages, such as lower household income, financial strain, and emotional distress [30, 31] - Food insecurity rates rose [32] - Unemployment led to the loss of health insurance for many individuals [32] | - Significant job loss occurred early in the pandemic, leading to high rates of food insecurity. - Lower eviction rates were observed, but there was an increased disconnect from healthcare services. - A greater need for internet access and devices emerged, particularly for children's distance education. | Technology use increased, including for virtual visits (e.g., Zoom), document sharing (e.g., Google Docs, Excel), and accessing services via government or other websites. |
| General Attitude towards Healthcare Providers | - Historical distrust of healthcare providers[33] - Some individuals feel that physicians treat them differently based on their SES.[34] |  | - Appreciate outreach workers for their companionship and ability to guide them step-by-step - Would like outreach worker to help determine goals - Looked at outreach worker as providing a “second opinion” regarding needs, but moreover to provide guidance to address priority needs |
| General Willingness to provide information | - Users may be willing to participate in baseline health, SDOH, and health informatics literacy surveys to identify gaps and strengths for outreach workers to address [35] - Concerns about privacy and data security are common [36] - Older adults are often less concerned about online privacy due to a lack of awareness regarding data security risks.[2, Ch. 6] - Informed consent mechanisms, explaining security considerations in plain language, are necessary when recommending technology to older adults.[2, Ch. 6] |  | - Participants prefer providing information independently, with outreach worker guidance as needed. - They are open to completing social needs assessments and additional surveys, but dislike revisiting previously answered questions frequently. - App-based surveys present challenges such as survey length, screen fatigue, and technical issues like unreliable phone connections. - Breaking surveys into smaller sections and offering technical support is recommended. - Providing alternative methods for non-smartphone users and using incentives, like gift cards, can increase participation. - Simplified language and instructions help mitigate health and digital literacy barriers. |
| Attitudes towards Goal Setting | Greater success in goal achievement is noted when community health workers assist in creating small, attainable goals [25-27] |  | - Users prefer setting realistic, manageable goals with the assistance of outreach workers, which helps them feel more supported in addressing their social needs. |
| DHT Use | | | |
| Existing Smartphone Experience | - Americans with lower incomes increasingly rely on smartphones. As of early 2021, 27% of adults in households earning less than $30,000 a year are smartphone-only internet users, meaning they own a smartphone but do not have broadband at home [18]. - 24% of adults with incomes under $30k do not have smartphones, though many can easily obtain access to one [18] - Smartphones are heavily used for employment and job-seeking [37] | - Most users have smartphones, but many are not proficient in using internet-based features independently. - A small but notable portion of users lacks reliable, consistent smartphone access, with some having limited data or no devices or data plans. Linguistic, educational, and other barriers are common. | - Users prefer to have a limited number of accounts on their smartphones. - Many seek assistance with downloading apps. - Email usage varies significantly, with some checking it daily, while others do so less frequently. |
| General Attitude Toward mHealth) | - Diverse, low-income communities have shown more interest in using mHealth apps, particularly for chronic disease management and overall health, compared to white, high-income communities [38] - Concerns about privacy and data security are common [39] | Not tracked | - Users often fear scams and worry that their personal information is being sold or monitored. - Concerns include long, unclear terms of service, privacy issues, and the potential for hacking. - While many are willing to provide email addresses and phone numbers for account recovery, they are wary of spam. - Saved passwords are convenient, but security concerns remain. - Users often rely on family members for help with technology and are generally nervous about using it, although they express a desire to learn. - Some users find automated messages or reminders annoying, while others benefit from them. |
| eHealth/mHealth usage | - Low-SES individuals are less likely to use eHealth compared to higher SES populations [40] - Technology is primarily used by low-SES individuals to understand symptoms and medical terms rather than to directly interact with the healthcare system [41] - Spanish speakers in low-SES communities are less likely to use eHealth than their English-speaking counterparts [33] | Portal for current EHR | - The COVID-19 pandemic increased the use of various technologies, including telehealth systems like Zoom, Google Docs, and others, though some found these systems frustrating and challenging to use. Issues included difficulties in account creation, password recovery, and address verification. - To manage health and wellness, reported use of: patient portal, WhatsApp (could be useful to connect with outreach workers and service organizations), Google/web search, WebMD, MyChart (to check medical history), Zoom (to speak with providers), Workout pro, church app, bible app - Some still have not used apps for health - They are willing to answer many questions in one sitting and appreciate clear icons and indicators in surveys. |
| Security Requirements | - Concerns about privacy and data security are common among users [42] - Some users express a desire for consent and knowledge of who accesses their data [33] - Older adults may be less concerned about privacy due to a lack of awareness regarding online data security issues [2] | HIPAA standards apply, and users are generally open to providing relevant health information but remain concerned about data privacy and security. | - Security concerns, particularly around hacking, are significant. Participants appreciate security questions with complex answers for added protection. - Experiences with spam and the fear of losing their phone contribute to apprehension. - Users find verification codes cumbersome and worry about their effectiveness in preventing unauthorized access. - Although they seldom read the terms of service, they feel obligated to agree to them to access services. |
| Platform (& Screen Resolution) | - Design should follow Nielsen’s heuristics: "visibility of system status, match between system and the real world, user control and freedom, consistency and standards, error prevention, recognition rather than recall, flexibility and efficiency of use, aesthetic and minimalist design, help users recognize, diagnose, and recover from errors, help and documentation" [43] - For older adults, designs should be simple with good color contrasts, large font sizes, and buttons for easy reading and clicking. Icons should be accompanied by text to aid understanding [2, Ch. 6] |  | - Users prefer all questions to fit on one screen without the need for scrolling. - Clear indications of survey progress are desired to reduce uncertainty. - Users appreciate clear indications of priority needs, such as color-coded scores or rankings. - A success message is valued as it reassures users that they are on the right track and instills confidence. |
| Co-user/ Support for mHealth use | One promising intervention is the use of community health workers (CHWs) to support patients in addressing social determinants of health and promote greater usage and understanding of health applications [33] | - Varying levels of ability among participants. - Clinical staff, outreach, and family members provide support to participants to access technology. | Many users are dependent on support of co-users |
| Motivation for Using the System and Specific Goals | Literature suggests that collective goal setting for addressing various SDOH issues is facilitated by social/outreach workers [25-27] | Household members do not access services through an electronic system with the organization; instead, they rely on public resources, with staff assisting them in navigating those resources based on their perceived needs. | - Users prefer selecting goals through the app at their own pace, allowing them to read and consider the information. However, they value in-person support for addressing questions. - Users see value in goal setting but express concern that some goals may not apply to their specific family situations. |

1. Covello S. A Review of Digital Literacy Assessment Instruments. IDE-712: Analysis for Human Performance Technology Decisions. 2010.

2. Smith CA, Keselman A. Consumer health informatics : enabling digital health for everyone. First edition ed. Boca Raton, Florida ;: CRC Press; 2021. ISBN: 0-429-80888-7.

3. Agness C, Murrell E, Nkansah N, Martin CM. Poor Health Literacy as a Barrier to Patient Care. The Consultant pharmacist : the journal of the American Society of Consultant Pharmacists. 2008;23(5). doi: <https://doi.org/10.4140/tcp.n.2008.378>.

4. Schillinger D. The Intersections Between Social Determinants of Health, Health Literacy, and Health Disparities. Studies in Health Technology and Informatics. 2020;269:22-41. doi: <https://doi.org/10.3233/SHTI200020>.

5. Towe VL, May LW, Huang W, Martin LT, Carman K, Miller CE, et al. Drivers of differential views of health equity in the U.S.: is the U.S. ready to make progress? Results from the 2018 National Survey of Health Attitudes. BCM Public Health. 2021;21(1):175. doi: <https://doi.org/10.1186/s12889-021-10179-z> <div data-component="share-box" style="margin: 0px; box-sizing: inherit; padding: 0px; color: rgb(51, 51, 51); font-family: Georgia, Palatino, serif; font-size: 18px; background-color: rgb(255, 255, 255);">.

6. Elgaddal N, Kramarow EA, Reuben C. Physical Activity Among Adults Aged 18 and Over: United States, 2020. National Center for Health Statistics, U.S. Centers for Disease Control and Prevention, 2022 Contract No.: Data Brief, Number 443.

7. Office of Disease Prevention and Health Promotion U.S. Department of Human and Health Services. High School Graduation. [cited 2024 November 22, 2024]; Available from: <https://odphp.health.gov/healthypeople/priority-areas/social-determinants-health/literature-summaries/high-school-graduation>.

8. National Center for Education Statistics U.S. Department of Education. Trends in High School Dropout and Completion Rates in the United States: 2018. 2018.

9. Office of Desease Prevention and Health Promotion U.S. Department of Health and Human Services. Food Insecurity. [cited 2024 November 22, 2024]; Available from: <https://odphp.health.gov/healthypeople/priority-areas/social-determinants-health/literature-summaries/food-insecurity>.

10. Economic Research Service U.S. Department of Agriculture. Food Insecurity in the U.S. - Key Statistics and Graphics. 2024 [cited 2024 November 22, 2024]; Available from: <https://www.ers.usda.gov/topics/food-nutrition-assistance/food-security-in-the-u-s/key-statistics-graphics/>.

11. Agency for Healthcare Research and Quality. ACCESS TO HEALTHCARE AND DISPARITIES IN ACCESS. National Healthcare Quality and Disparities Report [Internet]. Rockville (MD)2021.

12. Services OoDPaHPUSDoHaH. Housing Instability. [cited 2024 November 22, 2024]; Available from: <https://odphp.health.gov/healthypeople/priority-areas/social-determinants-health/literature-summaries/housing-instability>.

13. Joint Center for Housing Studies of Harvard University. The State of the Nation's Housing 2020. 2020.

14. Pew Research Center. Republican Gains in 2022 Midterms Driven Mostly by Turnout Advantage. 2023.

15. Office of Disease Prevention and Health Promotion U.S. Department of Health and Human Services. Poverty. [cited 2024 November 22, 2024]; Available from: <https://odphp.health.gov/healthypeople/priority-areas/social-determinants-health/literature-summaries/poverty#cit3>.

16. Shrider EA, Kollar M, Chen F, Semega J. Income and Poverty in the United States: 2020. United States Census Bureau; 2021 [cited 2024 November 22, 2024]; Available from: <https://www.census.gov/library/publications/2021/demo/p60-273.html>.

17. Chetty R, Matthew O.Kuchler, TheresaStroebel, JohannesHendren, Nathaniel Fluegge, Robert B.Gong, SaraGonzalez, Federico Grondin, Armelle Jacob, MatthewJohnston, DrewKoenen, MartinLaguna-Muggenburg, EduardoMudekereza, Florian Rutter, Tom Thor, Nicolaj Townsend, Wilbur Zhang, Ruby Bailey, MikeBarberá, PabloBhole, Monica Wernerfelt, Nils. Social capital I: measurement and associations with economic mobility. Nature. 2022;608:108-21. doi: <https://doi.org/10.1038/s41586-022-04996-4>.

18. Vogels EA. Digital Divide Persists Even as Americans with Lower Incomes Make Gains in Tech Adoption. Pew Research Center, 2021.

19. Ng AE, Adjaye-Gbewonyo D, Dahlhamer J. Lack of Reliable Transportation for Daily Living Among Adults: United States, 2022. National Center for Health Statistics, Centers for Disease Control and Prevention, 2024 Contract No.: Data Brief No. 490.

20. Weissman J, Pratt LA, Miller EA, Parker JD. Serious Psychological Distress Among Adults: United States, 2009-2013. National Center for Health Statistics, Centers for Disease Control and Prevention, 2015 Contract No.: Data Brief No. 203.

21. U.S. Bureau of Labor Statistics. A profile of the working poor, 2021. 2023.

22. Wardle J, Steptoe A. Socioeconomic Differences in Attitudes and Beliefs About Healthy Lifestyles. Journal of Epidemiology and Community Health. 2003;57(6):440-3. doi: <https://doi.org/10.1136/jech.57.6.440>.

23. Reshma P, Rajkumar E, John R, Allen JG. Factors influencing self-care behavior of socio-economically disadvantaged diabetic patients: A systematic review. Health Psychology Open. 2021;8(2). doi: <https://doi.org/10.1177/20551029211041427>.

24. LeRouge C, Austin E, Lee J, Segal C, Sangameswaran S, Hartzler A, et al. ePROs in Clinical Care: Guidelines and Tools for Health Systems. Seattle, WA: CERTAIN, University of Washington; 2020.

25. Craig SL, Bejan R, Muskat B. Making the Invisible Visible: Are Health Social Workers Addressing the Social Determinants of Health? Social Work in Health Care. 2013;52(4):311-31. doi: <https://doi.org/10.1080/00981389.2013.764379>.

26. Kangovi S, Mitra N, Norton L, Harte R, Zhao X, Carter T, et al. Effect of Community Health Worker Support on Clinical Outcomes of Low-Income Patients Across Primary Care Facilities: A Randomized Clinical Trial. JAMA Internal Medicine. 2018;178(12):1635–43. doi: 10.1001/jamainternmed.2018.4630.

27. Collins ME, Mowbray CT, Bybee D. Establishing Individualized Goals in a Supported Education Intervention: Program Influences on Goal Setting and Attainment. Research on Social Work Practice. 1999;9(4):483-507. doi: 10.1177/104973159900900405.

28. Partiwi N. Investigate the Effectiveness, Mechanisms, and Scalability of Community-Based Interventions Designed to Reduced Health Disparities. Advances In Healthcare Research. 2024;2(1):49-65. doi: <https://doi.org/10.60079/ahr.v2i1.371>.

29. Brakefield WS, Olusanya O, White B, Shaban-Nejad A. Social Determinants and Indicators of COVID-19 Among Marginalized Communities: A Scientific Review and Call to Action for Pandemic Response and Recovery. (1938-744X (Electronic)).

30. Masterson JM, Luu M, Dallas KB, Daskivich LP, Spiegel B, Daskivich TJ. Disparities in COVID-19 Disease Incidence by Income and Vaccination Coverage — 81 Communities, Los Angeles, California, July 2020–September 2021  2023 Contract No.: 72.

31. Ringlein GV, Ettman CK, Stuart EA. Income or Job Loss and Psychological Distress During the COVID-19 Pandemic. JAMA Network Open. 2024;7(7):e2424601.

32. Singu S, Acharya A, Challagundla K, Byarareddy S. Impact of Social Determinants of Health on the Emerging COVID-19 Pandemic in the United States. Frontiers in Public Health. 2020;8:406. doi: <https://doi.org/10.3389/fpubh.2020.00406>.

33. Liu P, Astudillo K, Velez D, Kelley L, Cobbs-Lomax D, Spatz ES. Use of Mobile Health Applications in Low-Income Populations. Circulation: Cardiovascular Quality and Outcomes. 2020 2020/09/01;13(9):e007031. doi: 10.1161/CIRCOUTCOMES.120.007031.

34. Arpey NC, Gaglioti AH, Rosenbaum ME. How Socioeconomic Status Affects Patient Perceptions of Health Care: A Qualitative Study. Journal of primary care & community health. 2017;8(3):169-75. PMID: 28606031. doi: 10.1177/2150131917697439.

35. Thomas-Henkel C, Schulman M. Screening for Social Determinants of Health in Populations with Complex Needs: Implementation Considerations. Center for Health Care Strategies, 2017.

36. Liu P, Astudillo K, Velez D, Kelley L, Cobbs-Lomax D, Spatz ES. Use of Mobile Health Applications in Low Income Populations: A Prospective Study of Facilitators and Barriers. Circulation: Cardiovascular Quality and Outcomes. 2020;13(9). doi: <https://doi.org/10.1161/CIRCOUTCOMES.120.007031>.

37. Pew Research Center. The Smartphone Difference. 2015.

38. Sharma S, Gergen Barnett K, Maypole JJ, Grochow Mishuris R. Evaluation of mHealth Apps for Diverse, Low-Income Patient Populations: Framework Development and Application Study. JMIR Formative Research. 2022;6(2):e29922. doi: <https://doi.org/10.2196/29922>.

39. Madden M. Privacy, Security, and Digital Inequality. Data & Society, 2017.

40. Williams C, Shang D. Telehealth Usage Among Low-Income Racial and Ethnic Minority Populations During the COVID-19 Pandemic: Retrospective Observational Study. Journal of Medical Internet Research. 2023;25. doi: <https://doi.org/10.2196/43604>.

41. Fitzpatrick L. The Time Is Now: The Case for Digital Health Innovation for the Poor and Underserved. *To the Point*: Commonwealth Fund; 2018. p. <https://doi.org/10.26099/VMDB-A964>.

42. Gordon A, Liu Y, Tavitian K, York B, Finnell SM, Agiro A. Bridging Health and Temporary Housing Services for Medicaid Members Experiencing Homelessness: Program Impact on Health Care Utilization, Costs, and Well-being. Journal of Health Care for the Poor and Underserved. 2021 Nov;32(4):1949-64. PMID: WOS:000721355700019.

43. Nielsen J, editor. Enhancing the Explanatory Power of Usability Heuristics. SIGCHI Conference on Human Factors in Computing Systems; 1994; Boston, Massachusetts: Association for Computing Machinery.
