# Supplementary material for: Evaluating the Needs and Characteristics of Individuals of Low Socioeconomic Status Using Digital Health Technology to Address Health-Related Social Needs: Mixed Methods Study With Patients and Care Providers
Source: JMIR Hum Factors. 2025 Sep 12;12:e69545. doi: 10.2196/69545 (PMC12475883; doi:10.2196/69545)
Supplement: Multimedia Appendix 2 [file humanfactors_v12i1e69545_app2.docx]

**Appendix 2. SDOH DHT Landscape Analysis**

| **Name** | **Modality** | **Locality** | **Developer** | **Website** | **Purpose** | **Description** |
| --- | --- | --- | --- | --- | --- | --- |
| **Both Government & Other Sponsored (n=7)** | | | | | | |
| Aunt Bertha/FindHelp.org | Website & Mobile App (Apple & Android) | Nationwide | FindHelp | https://www.findhelp.org/ | Assists individuals in finding local resources for essential services like food, housing, transportation, and employment | • FindHelp is a social determinants of health (SDOH) network platform.  • It connects individuals with local resources for essential services such as food, housing, transportation, and employment.  • Users can search anonymously for support tailored to their needs.  • The platform is designed to simplify access to critical resources and empower communities |
| HelpSteps | Website & Mobile App (Apple) | Massachusetts | Boston Children's Hospital | https://helpsteps.com | Connects individuals with children with Social Services | • HelpSteps is a social service referral system for Massachusetts, connecting individuals to resources like affordable housing, food, and employment.  • It aims to reduce health and social disparities by linking users to local health and human services.  • It is supported by organizations including the Boston Public Health Commission, Mass 211, and the Greater Boston Food Bank. |
| 211.org | Website | Nationwide | United Way Worldwide | https://www.211.org/ | Provides individuals across the U.S. and Canada with access to information on housing, food programs, healthcare, mental health support, employment services, and disaster relief | • 211 provides expert, confidential help to individuals in need.  • People contact 211 through calls, web chats, and text messages, seeking help for basic needs such as housing, food, transportation, and healthcare. |
| ACT.md | Web app | Nationwide | Activate Care | https://www.activatecare.com/ | Provides tools for managing complex care, tracking outcomes, and connecting individuals with community resources | • ACT.md equips providers with tools to view the full scope of a patient's needs and manage care collaboratively across community partners.  • Users can securely share tasks, messages, and data with health and social services partners through an embedded suite of tools.  • ACT.md integrates with EMR, allowing care teams to address unmet social needs and collaborate on care for unique populations.  • The platform supports cross-organizational care coordination for comprehensive patient management. |
| NowPow | Web app | Nationwide | Unite Us | https://nowpow.com/ | Provides personalized community referrals and care coordination tools, connecting individuals with the services they need to improve overall well-being and health outcomes | • FindRx, a NowPow platform app, places patients and caregivers at the center of self-care by offering screenings and a community resource directory.  • It builds personalized community referral networks, promoting equity and impactful partnerships.  • It supports various use cases, from self-serve to remote care, and integrates seamlessly with EHRs, HIEs, and care management systems.  • It ensures that referrals are integrated into routine workflows for diverse users. |
| Pieces | Web app | Nationwide | Pieces Technologies | https://piecestech.com/ | Provides AI-driven tools for clinical decision support, social determinants of health integration, and care coordination | • Pieces Predict uses artificial intelligence to rapidly identify at-risk patients in the Electronic Medical Record.  • It analyzes clinical, social, and economic determinants through predictive models and deep clinical algorithms.  • It connects care providers to actionable data, services to people, and caseworkers to information.  • It simplifies data input, management, storage, organization, and insight generation, supporting providers in helping patients. |
| WellSky Social Care Coordination | Web app | Nationwide | WellSky | https://wellsky.com/social-care-coordination/ | Connects healthcare providers, social services, and community resources | Network:  • WellSky facilitates partnerships between healthcare and community-based organizations to ensure clients receive necessary services.  • It works directly with community-based organizations to establish formal contracts that promote accountability.  • By building formal relationships within the network, WellSky ensures that the referral process is completed.  • The goal is to guarantee that clients receive the services they need through a reliable and accountable system.Platform:   • WellSky addresses inefficiencies in connecting clients to resources by maintaining a vetted and curated database of over 420,000 resources nationwide.  • It simplifies the referral process, ensuring clients are connected to appropriate services.  • Its goal is to reduce outdated or scattered information and make it easier for users to find vital resources efficiently. |
| **Government Sponsored Only (n=10)** | | | | | | |
| Your Texas Benefits | Mobile App (Apple) | Texas | TX Health and Human Services Commission | http://www.yourtexasbenefits.com/ | Assists people in Texas by providing access to and management of SNAP food benefits, healthcare benefits (Medicaid and CHIP), and TANF cash help | • The Your Texas Benefits app is for Texans who use SNAP, Medicaid, CHIP, or TANF benefits.  • Users can manage their cases from their phone, including checking benefit status, amounts, and renewal dates.  • The app allows users to send required documents, such as paychecks, by taking and uploading photos.  • Users can also set up alerts for important updates, report changes to their cases, and locate nearby offices.  • Features include setting up a new account, viewing benefit details, and renewing benefits easily. |
| ebtEDGE | Mobile App (Apple) | Nationwide | Fidelity National Information Services, Inc | https://www.fisglobal.com/en/ebtedgemobile | Assists individuals who depend on SNAP or TANF benefits to access their benefits | • The ebtEDGE mobile app simplifies the lives of users who rely on SNAP or TANF benefits.  • It allows users to easily view their benefit balances and transaction history. Users who have both SNAP and TANF benefits can access and manage both simultaneously. |
| Providers: EBT, debit, & more | Mobile App (Apple) | Nationwide | Propel Inc | https://www.joinproviders.com/ | Assists individuals in managing their benefits and income by providing a single platform to check food stamp balances, manage EBT, WIC, SSI, unemployment, and other benefits, and access a free debit account | • Providers (formerly Fresh EBT) is the top-rated app for checking food stamp balances and managing other benefits.  • Users can manage various income and benefits in one place, including EBT, WIC, Child Tax Credit, paychecks, tax refunds, disability/SSI, savings, and unemployment.  • The app also offers the Providers Card, a free debit account, for streamlined financial management.  • Providers are available in all 50 states and used by over 5 million people. |
| What I Need (WIN) | Mobile App (Apple) | California | OurCommunityLA | https://winwhatineed.net | Assists homeless, abused, and resource-insecure individuals by providing access to free resources across 12 categories in Los Angeles County | • WIN (What I Need) connects struggling or homeless individuals in Los Angeles County to free resources across 12 categories.  • It helps users find food pantries, shelters, healthcare, educational resources, crisis support, and more.  • WIN users can receive alerts about job fairs, connect to hotlines, and provide feedback on listed agencies.  • It serves vulnerable populations such as veterans, LGBTQ individuals, foster youth, and pregnant teens/adults. |
| myCOMPASS PA | Mobile App (Apple & Android) | Pennsylvania | Pennsylvania State Government | https://www.compass.dhs.pa.gov/home/#/ | Assists Pennsylvanians who have applied for or receive health and human services programs by providing easy access to their benefits, allowing them to manage applications, check status, renew benefits, upload documents, and report changes | • myCOMPASS PA is designed for Pennsylvanians who use health and human service programs, allowing them to access benefits on the go.  • It offers many features of COMPASS, including checking benefit details, application status, and renewal dates.  • Users can upload, send, and view required documents, and report changes to personal information like addresses and phone numbers.  • Tasks that previously required a visit to the county assistance office can be completed within minutes through the app. |
| ACCESS Florida | Website | Florida | FL Department of Children and Families | https://www.myflorida.com/accessflorida/index.html | Provides access to essential benefits like food assistance (SNAP), cash assistance (TANF), and medical coverage (Medicaid) in Florida | • The Florida Department of Children and Families' Public Assistance webpage provides information on programs like SNAP (food assistance), TANF (temporary cash assistance), Medicaid, and refugee services.  • It offers resources for applying, checking eligibility, and managing benefits.  • The webpage serves as a central hub for accessing public assistance, aiming to support the well-being of Florida residents in need. |
| Washington Connection - Your link to services | Website | Washington | Washington Connection | https://www.washingtonconnection.org/home/ | Helps individuals and families in Washington State find and apply for various public benefits and services | • Washington Connection provides a quick and easy way for families and individuals to apply for various services, including food, cash, childcare, long-term care, and Medicare Savings Programs.  • Those aged 65 or older, blind, or disabled can also apply for medical assistance through the platform. |
| Washington Healthplanfinder | Website | Washington | Washington Healthplanfinder | www.wahealthplanfinder.org | Helps individuals and families in Washington State find, compare, and enroll in health insurance plans, including Medicaid and other qualified health plans | • Washington Healthplanfinder offers healthcare coverage for children, parents/caretakers with children, pregnant women, and adults aged 18 to 64. |
| Benefit Finder | Website | Nationwide | US Government | https://www.benefits.gov/ | Helps individuals find and access federal benefits for which they are eligible, including healthcare, food assistance, and housing support | • The Benefit Finder questionnaire helps users identify benefits they may be eligible for.  • It directs users to the appropriate agency to apply for those benefits. |
| HHS.gov Social Services | Website | Nationwide | US Department of Health and Human Services | https://www.hhs.gov/programs/social-services/index.html | Provides information on various programs, including child welfare, family support, disability services, and services for older adults | • HHS oversees programs and services that improve the well-being of individuals, families, and communities.  • Programs are designed to provide resources and support across a wide range of social needs. |

** The descriptions presented in the Appendix were derived from developer materials.*
