# Supplementary material for: Evaluating the Needs and Characteristics of Individuals of Low Socioeconomic Status Using Digital Health Technology to Address Health-Related Social Needs: Mixed Methods Study With Patients and Care Providers
Source: JMIR Hum Factors. 2025 Sep 12;12:e69545. doi: 10.2196/69545 (PMC12475883; doi:10.2196/69545)
Supplement: Multimedia Appendix 3 [file humanfactors_v12i1e69545_app3.docx]

**Multimedia Appendix 3. Evidence Trace Table**

| **Theme** | **Features** | **Open Codes** | **Quotes** |
| --- | --- | --- | --- |
| User-Centered Design | Multilingual translation (English, Spanish, Creole) | Language barriers | "With filling out surveys and all that stuff, sometimes you need help with translation" |
|  |  |  | "With the NeighborhoodHelp families, sometimes language can be a barrier" |
|  |  | Consider language and culture | "We have to have Haitian Creole as a necessity, and there's probably even other languages that we could consider but I think English, Spanish, & Creole are top three" |
|  |  |  | "My concern being bicultural, Hispanic, is how [the questions] come across in Spanish and other languages" |
|  | Enable those with lower levels of education to use and learn from app | Lower educational literacy | "Some people may not even know or be educated enough to know exactly what you're trying to say. You know like sometimes they are people who haven't been to school for some reason or the other and they find it difficult in putting the words together or even know what they're doing." |
|  |  | Must consider users with lower digital literacy | The app should also be user friendly and intuitive. It should be designed so that regardless of the literacy level, the user can know how to use it. It should be simple and efficient. |
|  |  | Instructional training videos | Websites and videos and the diagrams like the wheel were helpful. |
|  |  |  | Video will be more helpful because at least you can see what it's all about, you will be speaking it, and yet they could see it. So I think they could understand better. If you just you know, if you share a diagram, for instance, there are a lot of people who may not be able to read, especially the older folks may not be able to read the diagram, but the video looking at the video, you know, they can understand, I think. |
|  | Integrate user-selected priorities with validated social risk factor assessment indications | Triad: Patient priorities, provider insight, feasibility of meeting need | So, the only thing I would say is definitely take into consideration three things I said earlier, which is how the patient or client values a certain area, you know, how the individual that's providing that resource values that certain area, and the ability to provide a resource for that certain area. So, the likelihood or the resources available to address that. I think those three levels should be considered when trying to figure out what is the most important as well as effective efficient area that we can kind of addressed |
|  |  | Integrate patient priorities | It is crucial to know the patient's priorities and perspectives. |
|  | Notifications/reminders suited to user status and planned actions | App reminders | "People might need a nudge from NeighborhoodHELP, the outreach worker [...] to say did you actually follow up" |
|  |  |  | "I'm focused right now in my life on being consistent with things and [apps] have helped me to do that. They give you each day and if you don't do it, you will see that notification on the top of your phone, so kind of reminding you" |
|  | Simple, step-by-step walkthrough | Step by step walkthrough: wording & explanations | You know, because they have such a team that, you know, they make everything, they do their best to make everything, you know, easy by walking you through step by step making you, you know, making sure that you understand details of it. |
|  |  | Straightforward simple flow walkthrough | Yeah, that would be great. It’s actually better because you’re talking about one specific thing. When you mix everything up, you’re like—the thing is that my time at home, I have a lot to do |
|  |  | Improve flow | "It would need to have like an easy flow for people to engage with, you know, if they don't have to keep scrolling" |
|  | Pre-filled responses | Leveraging existing information to reduce repeated collection of stigmatizing data | Okay, these are the areas that they need help, and they don't have to reintroduce themselves. So, like, you know, yeah, I have food insecurity, this, this and that, two semesters later, I still have food insecurity, you know, so it's, it's less victimizing or not victimized, but like traumatic or kind of like, self-esteem, I just see it as that way. |
|  | Avoid repetitive and lengthy data collection | Cannot be repetitive, Complete in phases (save & return later), Do not want to do things over and over again | Some people don’t want to end up doing the same thing over and over. If I do it on here, and once I get there, I got to do the shit all over again, answering the same stuff, what did I do that for? It’s a waste of time. Depending on if there would be other stuff in the office, I would say if they do go back to it, they should have the choice to go back to it on the computer or the phone, or to finish it at the place. Give them that option. |
|  |  | Managing Length of Intake, Complete in phases (save & return later) | We have to make sure that it’s brief. Surveys that are too long can be really boring, and you only have so much time, so it’d really be helpful if you could save it and then come back to it. |
|  |  | Complete in phases (save & return later) | Not to provide all the questions at once, because that would make some people overwhelmed. So we should do it in stages |
|  |  |  | if they're too long, you know, we should give people the opportunity to save their answers and allow them to come back to them |
|  |  |  | Probably you can do it in parts. Not putting all of it probably, maybe it is 10 questions and you do it like in series |
| Efficient Solution-Based Assessment of Social Risk/Need | Application has comprehensive coverage of the spectrum of SDOH domains | Holistic (including cultural and social) overview of social needs | Get a holistic view of the social needs of a client rather than just what specific and at the moment, you want some background and cultural background as well. |
|  |  | Ask about all domain areas | I think the information gathered is very crucial. And it does cover a wide domain of functions and social determinants that I think should remain like the content itself of what's being asked. |
|  | Validated social risk factor assessment results inform user of social risk areas. | Would want to see need areas calculated by the system | So would it be useful for you to see the results of what areas might need help or might need the high priority area to work on. Interviewee  Um, yeah, that'd be helpful because it helps make somebody like us or like me to be able to kind of help narrow it down |
|  | Straightforward process for selecting and using a single service to meet social need | Expects to select a specific area and see resources | You said you would choose to assess your needs, because you said you will you need to see what your needs are so that we would know where to direct you to |
|  |  | Consolidated view/Summary of current services in use | That's a really good idea. And so you need, I'm going to ask you that, if you can go ahead, because I think there's some there's a lot that you're saying with what you just indicated with basically a limited view, for those people that are not going to be continuing with the system. If you could go ahead and add to that in terms of some of the things that you indicated, because some of your colleagues are saying, yeah, yeah, we like that idea of specifically what you think would be on that limited view, that would be really important. Participant 9: referrals being used |
|  |  | Add option to bookmark/save to list of services | You can include anything. I would say that if you guys could add one thing, maybe an option to bookmark, or possibly save it to like a list of bookmark resources, so if multiple things might apply for what I need, maybe I could save them. I mean, I can always write it down or do something else. But I guess as far as like maybe a convenience feature, add that. The review feature is nice, that's very nice to have. |
|  | Comprehensive service list organized based on user's social needs | Add more details on initial service list screen | So in other words, if you've identified you have a food need, you want the list of services to say that this service helps to meet your food need. |
|  |  | Order list according to need with most popular/best fit | Or, or be like, maybe if the list looks like, it's too long. So I would say they should have like, you know, choose the best one and just put the best one in there. |
|  |  | Determine if service is close to their location | In certain areas or areas around me |
|  |  | Like that they see eligibility requirements | Is that important to you, to get the eligibility information for the service right away?  Participant: Yeah, to know if I'm eligible for it or not. Absolutely, that'd be wonderful. There's no guessing into it, like I'm either eligible or not. That'd be great if that’s possible. |
|  | Social risk assessment results and service progress can be shared with user's healthcare/wellness network | Integrating Thrive with Additional Systems in Use and Service Organizations | Participant: I would like it to serve as a bridge from portal to our EMR |
|  |  | Would want to discuss results with outreach worker after having reviewed them on their own, Allow client to determine need level, Allow client to determine challenge/goal level | Yes, I think we should put input, but we don't want to, we don't want to persuade them. Or, you know, we don't want them to feel pressure into what we feel is their needs, because then it wouldn't be as honest. So maybe we can, we can probably help them just then explaining because or even if you put on the app, you know, like maybe another... I mean, priorities are pretty straightforward to us. But if someone might not use that word, or if they have a language barrier, you might want to just say something like, Okay, what, what's most important to you, you know, but still have priorities, but then also, what's most important to you so then that way, it's just straightforward and to the point. And then yeah, and then after they get them doing their, you know, once they they're able to organize it, then you could come in afterward like PARTICIPANT 24 said, |
|  |  |  | we want to get a way to meet the client where they're at and to find out what their social needs are and which of those social needs are relevant to how we can help them. So, it might be helpful to get a background of social needs, but some of those social needs may not be relevant at the moment or might not be as relevant to the particular task or challenge at hand if that makes sense kind of thing. So, understanding the social needs in their broader context and then which are particularly relevant to the situation at hand. |
| Education & User Feedback (Reviews and Ratings) | Explanations to improve existing digital health literacy and understanding of SDOH implications on health outcomes | Information is helpful | "I think it is very helpful because [...] a lot of people don't know [...] how important it is they have insurance. Or some people might think it's too expensive and it's not. The point of having it. The screening. Why you need to keep up with your flu shot and so forth." |
|  |  |  | "Would that be of interest to you, to learn more about these different areas and how it could affect your quality of life and health? Yes, that would be good" |
|  |  | Value creation- explain reasoning behind topics/questions | If you don't understand why somebody would be asking, you're not going to answer it |
|  |  |  | It is really important that you understand why they're asking you certain questions |
|  |  | Message more than common knowledge and basic definition | "If someone didn't get health insurance first, but you indicated that, and then it's more information than they're given better insight like "OK, this might be helpful" |
|  |  | Patient education | access to knowledge itself, a lot of the services that are out there, if they are available to them, a lot of situations, they just don't have knowledge of it, or don't know how to navigate themselves through the process of getting those services. So it feels more readily accessible for our households, which, you know, really can vary in education level from, you know, low high school, to graduates to professionals. Something that's universally easy to access, I think is important, especially the access of knowledge itself, because that would cover a lot of situations, that's usually a big part of an outreach specialist job, it's informing our households how to reach or how to get access to those tools, certain resources within the community, or a realistic distance of their community. Because given the limitations of our households, depending on what it is. Transportation, for instance, was touched on a little bit earlier ago, that could be a huge barrier for individuals to practically perform their daily activities, which can hinder their ability to, to maybe go out of their way to acquire that knowledge. |
|  | User and outreach worker feedback on past service experience | Want to give feedback to share their experience with others / want to share ideas for improvement | Yes, when I use service and they use the surveys, I usually use them. I let them know if it is good. Because I want other people to know. I want to share my information because it might help somebody else. Yes, I'm good for that, for sharing information. |
|  |  |  | Yes, I would prefer to leave the comments, because at least the comments have people to know who you're dealing with and what to expect based on the service that was given to you by the person or the association, so to speak. |
|  |  |  | For me, no because one of the things I understand is when they asked me for my feedback is like the other people that need to see those things and to understand that they will help. You know, when I give my feedback it’s like, I want other people to know that, for example, the university program really helps so this is what I was going through, and they helped me with that. So great program and stuff like that. If there's something that I didn't like I just put it there, but I make sure that it's in a positive way so that they can find a way to correct it and make it better. |
|  |  | Uses ratings to determine quality of service | Do you see yourself Want to see ratings on the services?  Interviewee  Um, yeah, that'd be good. Yeah, that'd be good. Because I don't want to go to no place where it's like a one star. And they'd be like, Oh, my God, this service is bad. And, you know, and I could go to somewhere else. And they'd be like, hey, you know, you should come here the services great. You're know, you're pretty much in and out is no difficult situation, no long lines, or whatever the case may have been. So good feedback is the best feedback. |
| E-caring and Support | Use of empathetic and cooperative tone and language | App conveys sensitivity & empathy | You just have to know how to word it and how to construct the questions so they wouldn't be awkward, but I think certain questions people prefer to answer it on–certain things like that if they can answer it online then just say it to a person. |
|  |  | Developing a good relationship trust with the client | One thing that might have to be considered is how do we build that that relationship building in the process? 32:57 Participant 19: Yes, okay, it's making more sense in my head. Meaning that if they trust the system, and they trust, you know, the overall program, I guess, they will be willing to open up and provide their social determinants, such as the type of food they eat, the lifestyle they have, and so on. So I would say maybe make an app that is people friendly, and a type of vocabulary that they can relate to, I would say, |
|  |  | Gives reassurance/affirmation without having physical interaction with outreach worker | I mean, it's very, like, reaffirming or gives me the reassurance that interacting with my phone, even though I'm not physically talking to someone, just gives me more of a sense, well, okay, they put a lot of effort into making sure that me as the app user knows that they really want to take my feedback into consideration. |
|  |  | Developing a good relationship/trust with the client | And we also have a comment about health literacy needs to be considered as part of gathering information that that's a key point. Does anyone want to add anything else? 33:38 Participant 18: Yeah, culture plays a role in that too, because not everybody wants to open up to you the first time. But if it's an app, they know they can go there express themselves, like they are the one filling it out would also help as well. Because in some cultures, people tend to close when they don't know you, once you build a rapport and you inform them of what the purpose of the thing is, once they get to use it themselves, they are able to open up more to it. |
|  | Focus on empowering individual to be independent yet supported as they work through the app | Makes you feel like you're part of the solution | Do you like the idea of being able to set goals related to your priorities? Do you take time to do that? Do you find it useful? Is it just a waste of time? Participant: No. I like that, and the reason I like it is because I have the option of saying how we’re going to—I can be a part of what’s done. I can’t think of the word right now. Moderator: You’re part of the solution? Participant: That’s the word. Part of the solution rather than you just telling—Community Program won’t just tell me, “OK, I’m going to find a contact, and they’re going to do so and so, and so” I can be a part of what’s being done. Moderator: It sounds like you like it. It energizes you to be part of the solution. Participant: Yes. I like that. |
|  |  | Encouraging messages - find them helpful | "also a voice that can provide encouragement along the way, in addition have different messages like success or you know kind of here's how I'm progressing could be helpful" |
|  |  | Feeling of accomplishment | When it came to motivational messages, like a message saying success, you finished this particular survey, you said that that makes you feel great, so make sure that we keep those types of messages. |
|  | Service progress tracking | Would update progress in Thrive app | Would you be willing to let us know, hey, I received the service, I have not applied to it yet? Is that something that you would be okay sharing on the app? Participant: Yeah, I think that should be a part of it. Because I think that definitely, for me, I'm the king of procrastination, so it'll just keep me a little bit more accountable. |
|  |  | Would provide rating of service organization, Want to give feedback to share their experience with others | Moderator: And that's something else to close the loop and part of status is to determine who's actually used to service. So would you be willing as part of the goal and the plan that you set up to go back in and say yes, I you know, I tried to contact the service. I used them.  Participant: Yeah. |
|  | Basic IT skill support | Chat Service | Then if you had something like a chat that you can talk to someone, like a chat. You ask them questions, and they give you feedback. Something probably more like that for seniors. You know what I'm saying? That they can chat with that person also besides the app. Some seniors are more advanced than others. You know what I'm saying. So, maybe they can have that option. The chat group. Because I know I've used that on several occasions when I've had a problem with anything. I could go in there and chat. They help me find certain things that I need to find through the chat. I find them very helpful. They explain to me how to do it and this and that. So, I think something like that would be very helpful. |
|  |  |  | When I mean, guiding is like, okay, like, let's say, I don't understand, and I will click on a, let's say, there's a person there, I will click on that person to ask him what kind of question was, or I can type it. |
|  |  | Provide the option to complete on their own or with assistance | I think what should be added on is like giving them the option to either do it themselves or have somebody assist them. |
| Trust, Privacy, Security | App displays explicitly emphasize trust | Ensure privacy and confidentiality | And see if it’s invasive in my life. If it’s going to be invasive, I’m not going to mess with it. |
|  |  |  | I’m concerned because it’s a lot that has happened with these big companies, with individuals, regular people. You would think the big companies would take the precaution because they say, “Your privacy” and all that, but yet stuff still happens. You have hackers that are really good at what they do. I am concerned. It’s sad that people go through life having to take from others and not knowing their struggles, they’re not warned about the more struggle that they’re putting on people. I am worried about hackers, that’s something big. |
|  | High level of privacy, data security | Sensitivity to providing contact method due to spammers | "If I put my phone number, it depends on what it's going to be used for because sometimes you sign up with your phone number and then you get all these crazy text messages or you get all of these emails: |
|  |  |  | "Trusting the fact that certain people or certain apps and certain things you sign up for will say "This is 100% confidential" and then the next thing you know, you have 99 phone calls in a day, 75 emails in an hour" |
|  |  |  | "Once so much stuff gets in the way or I get so many phone calls, I get so many emails and other things that have nothing to do with Thrive, I'll be disinterested again" |
|  |  | Worry about hacking | "Putting it on black and white right there because everything can be hacked into. And it is used against you later on" |
|  |  | Fraud/scam concerns | I read some that were very straight to the point, and then I’ve read that some that was, what the hell is all of this? Those make me feel like they are trying to ease something in that you’re going to consent to. It’s kind of hard to trust. I have trust issues. |
|  |  |  | Sometimes I don’t log in to those apps which they send on Google. Gmail and all that. I just avoid those apps, because they can be scam, fake. |
|  | User control and understanding of data collected by app | Concerned over whether account had been deleted | Moderator: What went through your mind when this was happening? Participant: By now I’m used to it. The only thing I was worrying about was if my account had been deleted, that’s all. |
|  |  | App should ask permission before sharing information to others | privacy policy you said it should be a sentence that says, we respect your privacy, your information is secure, and should you choose, you would need to let us know by selecting what you would like to share. |
|  |  | People have different priorities / Optional questions | "Everybody has different needs and what I may think is not important, may be important to somebody else. So it'd be left up to the person if they want to answer such questions or not." |
|  |  | Hesitancy towards answering questions that are too personal | Wherever your needs areas are. What are your thoughts about filling out the questionnaire what's called an intake on your phone? Participant: Asking personal stuff, I guess I'll have to answer right? In order for them to find out. What could be wrong, right? I guess I have nothing against that. You know? (some hesitation in intonation) |
|  |  |  | I guess questions–the day-to-day activity questions, certain health-wide questions, certain questions when it comes to health that is common. I think when it comes to your reproductive health or your sexual life, a lot of people don't want to talk about it; a lot of people don't want to answer certain questions. |
|  |  |  | It depends, because there has to be more like, question now. Like, I'm, like, not too personal, but it's personal, who I would want to feel comfortable sharing. |
|  |  | Nosy intake questions but understands the need to answer them to get help | Yes, um, at first, it be a little uncomfortable, because you're like, Oh, my God, these people are nosy, but you can say stop. So it's like, you know, if I need the help, you know, I have to actually give them some type of clue or what I need, you know, they're not just gonna say, she need helped with this. No, it doesn't really like that. So it's kind of like a give and take situation. Like, I'm giving you something where you could give me something back, like, I'm giving you information about me, and you're giving me some way that I can be held. So it's like a give or take. |
|  |  | Potentially embarrassing questions are better answered on a phone than in person | You know sometimes, certain things people don't like to answer in person. Certain things people prefer just doing it on the app and be done with it; certain things are embarrassing to answer to. It's a slippery slope there. For me, I have no issues, but certain questions are uncomfortable with sometimes, but I would answer it, but a lot of people, I don't know. |
|  |  | Sensitivity to disclosing immigration status | For undocumented clients, they would need reassurance that info is confidential and will protect them from immigration |
|  |  | Sensitivity to disclosing employment/income | I would actually put no because it—that question can lead you to how much it is that you make. That may be something private that someone doesn’t want to discuss on a question. |
