# Supplementary material for: Evaluating the Needs and Characteristics of Individuals of Low Socioeconomic Status Using Digital Health Technology to Address Health-Related Social Needs: Mixed Methods Study With Patients and Care Providers
Source: JMIR Hum Factors. 2025 Sep 12;12:e69545. doi: 10.2196/69545 (PMC12475883; doi:10.2196/69545)
Supplement: Multimedia Appendix 4 [file humanfactors_v12i1e69545_app4.docx]

**Multimedia Appendix 4. DHT User Characteristics from Screening Survey**

DHT User Characteristics including Frequency of Internet Use, Means of Internet Access, Self-Rated Internet Skills, Frequency of Using Smartphone Apps, and Previous DHT Use.

| **Demographics** | **Number of Participants (%)** | |
| --- | --- | --- |
| **Frequency of Internet Use** |  | |
| (Almost) every day | 21 (81%) | |
| (Almost) never | 1 (4%) | |
| About 1 day a week | 2 (4%) | |
| Several days a week | 3 (11%) | |
| **Means of Internet Access** |  | |
| Mobile phone | 8 (31%) | |
| Mobile phone, laptop, computer at work | 1 (4%) | |
| Mobile phone, laptop, personal computer at home, public computer | 1 (4%) | |
| Mobile phone, laptop, personal computer at home, tablet | 2 (8%) | |
| Mobile phone, laptop, personal computer at home, tablet, computer at work | 2 (8%) | |
| Mobile phone, laptop, personal computer at home, tablet, computer at work, public computer | 2 84%) | |
| Mobile phone, laptop, tablet | 2 (8%) | |
| Mobile phone, laptop, tablet, computer at work | 1 (4%) | |
| Mobile phone, laptop, tablet, computer at work, public computer | 1 (4%) | |
| Mobile phone, laptop, tablet, public computer | 1 (4%) | |
| Mobile phone, Personal computer at home | 1 (4%) | |
| Mobile phone, tablet | 4 (15%) | |
| **Self-Rated Internet Skills** |  | |
| Average | 9 (35%) | |
| Excellent | 5 (19%) | |
| Good | 8 (31%) | |
| Poor | 2 (8%) | |
| Reasonable | 2 (8%) | |
| **Frequency of Using Smartphone Apps** |  | |
| (Almost) every day | 21 (81%) | |
| (Almost) never | 1 (4%) | |
| Almost 1 day a week | 1 (4%) | |
| Several days a week | 3 (12%) | |
| **Previous DHT Experience** | **No** | **Yes** |
| Search for information on health and wellness | 4 (15%) | 22 (85%) |
| Search for help with a basic need like food, shelter, or health care services | 7 (27%) | 19 (73%) |
| Schedule an appointment with their health care provider or community health worker | 11 (42%) | 15 (58%) |
| Read posts on a health-related forum or social media website | 7 (27%) | 19 (73%) |
| Have a video visit with a healthcare provider | 5 (19%) | 21 (81%) |
| Read a healthcare review of a healthcare provider (like a doctor) or facility (like a clinic) | 10 (38%) | 16 (62%) |
| Ask a question of your health care provider or community health worker | 7 (27%) | 19 (73%) |
| Monitor Disease Symptoms | 10 (38%) | 16 (62%) |
| Share personal medical information with others | 20 (77%) | 6 (23%) |
| Log on to your own electronic medical record or patient portal | 12 (46%) | 14 (54%) |
| Post a health care review of a health or social service provider or facility | 24 (92%) | 2 (7.7%) |
| Complete forms or surveys from your healthcare or social service provider on a computer or phone | 6 (23%) | 20 (77%) |
| Post a message on a peer support forum or social media website | 18 (69%) | 8 (31%) |
| Use a health-related mobile phone app | 12 (46%) | 14 (54%) |
